# Uncovering the genetic architecture of pungency, carotenoids, and flavor in *Capsicum chinense* via TWAS-mGWAS integration and spatial transcriptomics

**DOI:** 10.1093/hr/uhaf243

**Published:** 2025-09-15

**Authors:** Umesh K Reddy, Krishna Sai Karnatam, Alicia Talavera-Caro, Carlos Lopez-Ortiz, Kang-Mo Ku, Subramanyam Reddy Chinreddy, Sahithi Ramireddy, Purushothaman Natarajan, Virender Kumar, Sai Satish Kadiyala, Prapooja Somagattu, Ritik Duhan, Nagamani Balagurusamy, Vagner A Benedito, Donald A Adjeroh, Padma Nimmakayala

**Affiliations:** Department of Biology, Gus R. Douglass Institute, West Virginia State University, Institute, WV, USA; Department of Biology, Gus R. Douglass Institute, West Virginia State University, Institute, WV, USA; Department of Biology, Gus R. Douglass Institute, West Virginia State University, Institute, WV, USA; Department of Biology, Gus R. Douglass Institute, West Virginia State University, Institute, WV, USA; Department of Plant Biotechnology, College of Life Sciences and Biotechnology, Korea University, Seoul 02841, Republic of Korea; Department of Biology, Gus R. Douglass Institute, West Virginia State University, Institute, WV, USA; Department of Biology, Gus R. Douglass Institute, West Virginia State University, Institute, WV, USA; Department of Biology, Gus R. Douglass Institute, West Virginia State University, Institute, WV, USA; Department of Agriculture Food and Resource Sciences, University of Maryland Eastern Shore, Princess Anne, MD 21853, USA; Department of Biology, Gus R. Douglass Institute, West Virginia State University, Institute, WV, USA; Department of Biology, Gus R. Douglass Institute, West Virginia State University, Institute, WV, USA; Department of Biology, Gus R. Douglass Institute, West Virginia State University, Institute, WV, USA; Department of Biology, Gus R. Douglass Institute, West Virginia State University, Institute, WV, USA; Laboratorio de Biorremediación, Facultad de Ciencias Biológicas, Universidad Autónoma de Coahuila, Torreón 27275, Coahuila, México; Department of Agriculture Food and Resource Sciences, University of Maryland Eastern Shore, Princess Anne, MD 21853, USA; School of Animal and Food Systems, West Virginia University, Morgantown, WV, USA; Lane Department of Computer Science and Electrical Engineering, West Virginia University, Morgantown, WV 26506, USA; Department of Biology, Gus R. Douglass Institute, West Virginia State University, Institute, WV, USA

## Abstract

*Capsicum chinense* (habanero pepper) exhibits substantial variation in fruit pungency, color, and flavor due to its rich secondary metabolite composition, including capsaicinoids, carotenoids, and volatile organic compounds (VOCs). To dissect the genetic and regulatory basis of these traits, we conducted an integrative analysis across 244 diverse accessions using metabolite profiling, genome-wide association studies (GWAS), and transcriptome-wide association studies (TWAS). GWAS identified 507 SNPs for capsaicinoids, 304 for carotenoids, and 1176 for VOCs, while TWAS linked gene expression to metabolite levels, highlighting biosynthetic and regulatory genes in phenylpropanoid, fatty acid, and terpenoid pathways. Segmental RNA sequencing across fruit tissues of contrasting accessions revealed 7034 differentially expressed genes, including *MYB31*, *3-ketoacyl-CoA synthase*, *phytoene synthase*, and ABC transporters. Notably, *AP2* transcription factors and Pentatrichopeptide repeat (PPR) emerged as central regulators, co-expressed with carotenoid and VOC biosynthetic genes. High-resolution spatial transcriptomics (Stereo-seq) identified 74 genes with tissue-specific expression that overlap with GWAS and TWAS loci, reinforcing their regulatory relevance. To validate these candidates, we employed CRISPR/Cas9 to knock out *AP2* and *PPR* genes in tomato. Widely targeted metabolomics and carotenoid profiling revealed major metabolic shifts: *AP2* mutants accumulated higher levels of β-carotene and lycopene. In contrast, *PPR* mutants altered xanthophyll ester and apocarotenoid levels, supporting their roles in carotenoid flux and remodeling. This study provides the first integrative GWAS–TWAS–spatial transcriptomics in *C. chinense*, revealing key regulators of fruit quality traits. These findings lay the groundwork for precision breeding and metabolic engineering to enhance nutritional and sensory attributes in peppers.

## Introduction

Chili peppers, an important crop in the Solanaceae family, belong to the genus *Capsicum* and include at least 38 species. Five of them are domesticated: *Capsicum baccatum, Capsicum chinense, Capsicum frutescens, Capsicum annuum,* and *Capsicum pubescens* [1]. Their global production is 38 million tons across nearly two million hectares [2], underscoring their global significance. Among them, *C. chinense* (Habanero peppers) and *C. annuum* cultivars are in high demand in European and North American markets for their superior quality, high pungency, and exceptional flavor [3]. Habanero peppers are rich sources of bioactive compounds and minerals, contributing to their nutritional value and sensory attributes crucial for consumer acceptance [4]. Additionally, this species is characterized by its great diversity in fruit pungency levels, aroma, and color, indicating a wide genetic diversity [5]. Therefore, knowledge of the genetic basis of fruit pungency, flavor, and color variation in this species is essential for successful conservation and its use in breeding programs.

The main quality parameters of pepper fruits are color, pungency, and aroma [6]. Metabolites in *Capsicum* fruits are synthesized through diverse pathways, influencing nutritional qualities, fruit development, and consumer perception via taste and aroma [7]. Variations in metabolite concentrations significantly impact traits, such as color and flavor [8]. Recent advances in multi-omics data for an ever-increasing number of plant species, including high-throughput metabolomic, transcriptomic, and genomic platforms, have identified novel compounds linked to gene expression and elucidated key enzymes and regulatory factors in metabolic pathways for the highly diverse plant metabolome, complementing more traditional genetic and biochemical approaches [9–11]. In addition, they established a correlation between genotype and metabolite composition, clarifying the genetic architecture of complex biochemical pathways, such as the accumulation of secondary metabolites in plants, many of which are highly valuable for the human diet and the production of plant metabolite-derived medicines [12].

Genome-wide association studies (GWAS) and transcriptomic analyses are powerful tools for identifying significant loci associated with specific traits [13]. These methods utilize single-nucleotide polymorphisms (SNPs) and phenotypic variations, as well as gene expression profiles [14, 15]. Transcriptome-wide association studies (TWAS) have become a powerful approach for identifying genes underlying complex traits in crops by integrating gene expression profiles with phenotypic data. Unlike traditional genome-wide association studies (GWAS), which often struggle with the confounding effects of linkage disequilibrium (LD) decay and population structure in many crop species, TWAS can pinpoint causal genes more precisely and is robust even when using expression data from non-target tissues or developmental stages [10, 16]. In soybean, for instance, TWAS has identified both known and novel genes affecting traits, such as pod color and flowering time, with some findings validated through genome editing [10]. In rice, TWAS has uncovered regulatory networks involving transcription factors and epigenetic regulators that control panicle architecture [17]. These studies demonstrate that TWAS, particularly when combined with expression quantitative trait loci (eQTL) mapping, significantly accelerates the discovery of functionally relevant genes for crop improvement [16, 17].

Spatial transcriptomics is increasingly recognized as a crucial tool in crop research, as it enables the simultaneous quantification and localization of gene expression within intact plant tissues, thereby preserving the spatial context that is lost in single-cell or bulk transcriptomic approaches. This technology has been adapted for plant systems, overcoming challenges, such as rigid cell walls and high levels of secondary metabolites, and has demonstrated high specificity and accuracy in mapping gene expression to precise tissue regions [18]. However, [19] full-spectrum spatiotemporal transcriptome of eight chili pepper tissues at five growth stages was reported, with primary emphasis on developmental processes. By providing detailed spatial maps of gene activity, spatial transcriptomics has revealed the regulatory networks underlying key developmental processes, such as root regeneration in poplar, where it identified distinct anatomical regions and gene clusters involved in cytokinin and auxin signaling [20]. Furthermore, spatial transcriptomics provides a new perspective on plant growth, stress responses, and trait development by revealing localized gene expression patterns that drive phenotypic diversity and enabling the identification of tissue-specific biomarkers for crop improvement [20, 21]. The current study aims to explore phenotype–genotype associations through an integrated genome and transcriptome-wide association study and to construct a fruit-specific spatial transcriptome of contrasting *C*. *chinense* phenotypes. This approach generated a valuable resource for investigating single-nucleotide polymorphisms, gene expression, and regulatory networks to elucidate the genetic mechanisms governing fruit quality, including color, flavor, and pungency in *C. chinense* fruits, thereby promoting pepper improvement efforts.

## Results

### Phenotypic diversity in capsaicinoids, carotenoids, and flavor volatiles among *C. chinense* accessions

This study utilized diverse *C. chinense* accessions (Table S1) to analyze capsaicinoids, carotenoids, and volatile and nonvolatile flavor compounds. The ranges of concentrations for all metabolites are presented in Table 1 and Fig. S1, and complete phenotypic data for each *C. chinense* accession, including all metabolites quantified, are provided in Tables S2–S4. Capsaicinoids showed relatively high concentrations, with capsaicin and dihydrocapsaicin measuring 28.04 mg/g and 10.089 mg/g, respectively. The accessions with the highest capsaicinoid concentrations were Bhut Jolokia, Naga Morich, and the PI 497981 group, while those with the lowest concentrations were PI 653676, PI 543193, PI 653677, and Grif 9281. Carotenoid content was significantly high in the population, with α-carotene, β-carotene, capsanthin, and zeaxanthin concentrations at 277.77, 1321.66, 294.74, and 247.71 μg/g, respectively. The highest carotenoid concentrations were found in accessions PI 260522, PI 215736, PI 593929, and PI 241669, whereas the lowest concentrations were observed in PI 315028, PI 441629, PI 441620, PI 485593, and PI 257137.

**Table 1:** Concentrations of capsaicinods, carotenoids and flavor compounds in accessions of *C. chinense* used in the study

Capsaicinoids	mg/g	Mean
Capsaicin	0.01–28.04	2.11
Dihydrocapsaicin	0.00–10.09	0.56
**Carotenoids**	**μg/g**	**Mean**
α-carotene	0.11–277.77	43.38
β-carotene	0.67–1321.66	174.67
Capsanthin	1.13–294.74	57.37
Zeaxanthin	1.21–247.71	59.55
**Flavor**	**ng/g**	**Mean**
2-hexenal	13.8–7052.29	1028.73
4-methylpenthyl 2-methylbutanoate	0.03–886.1	282.767
4-methylpenthyl 3-methylbutanoate	5.01–8518.39	1017.57
4-Methylpentyl 4-methylpentanoate	0–61.376	5.30

For the flavor metabolites, the concentrations were generally similar across all measured compounds: 2-hexenal (7052.29 ng/g), 4-methylpenthyl 2-methylbutanoate (886.1 ng/g), 4-methylpenthyl 3-methylbutanoate (8518.39 ng/g), and 4-methylpenthyl 4-methyl pentanoate (61.376 ng/g), The highest concentrations of these flavor metabolites were found in accessions PI 281305, PI 543193, PI 439418, PI 260524, and PI 260466. In contrast, the lowest concentrations were identified in accessions PI 438629, PI 640902, and PI 441634.

The Shapiro–Wilk test confirmed that both capsaicinoids exhibit no significant deviation from normality, with values of *w* = 0.93565 for capsaicin and *w* = 0.86025 for dihydrocapsaicin. The Shapiro–Wilk test indicated that β-carotene, zeaxanthin, and capsanthin had the lowest normality values (0.7787, 0.8952, and 0.91052, respectively), while α-carotene had the highest (0.92314, respectively), suggesting a deviation from the normal distribution. Flavor compounds had a slightly skewed distribution. Overall, the distribution and normality analysis in *C. chinense* populations highlighted distinct patterns for capsaicinoids, carotenoids, and flavor compounds, with normality tests providing insights into the distribution approximations across different metabolites.

### SNP filtering and association mapping

**Figure 1:**
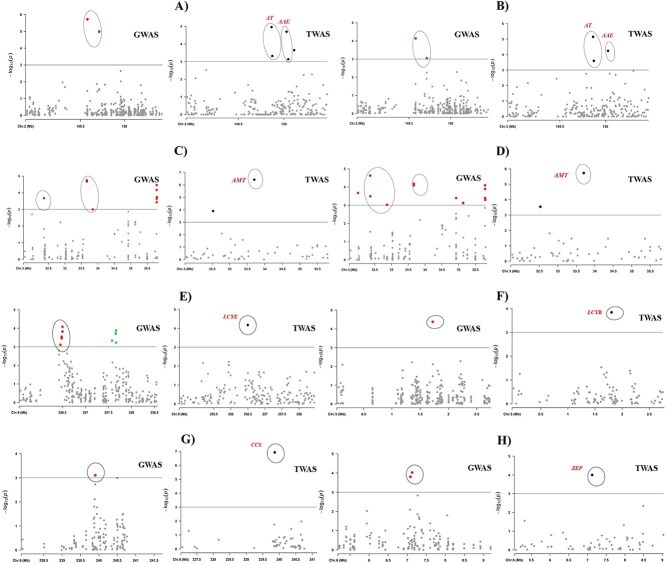
Genome-wide association studies (GWAS) and transcriptome-wide association studies (TWAS) reveal genetic and expression-based associations for key metabolic genes involved in capsaicinoid and carotenoid biosynthesis in *C. chinense* fruits. Panels A and B correspond to *Pun1* (Acyltransferase 1, *AT3*) associated with capsaicin and dihydrocapsaicin, while panels C and D depict associations for *Pun2* (Aminotransferase, *pAMT*) with the same metabolites. Panels E through H display loci linked to carotenoid biosynthesis, including *LCYE* (Lycopene ε-cyclase), *LCYB* (Lycopene β-cyclase), *CCS* (Capsanthin/Capsorubin synthase), and *ZEP* (Zeaxanthin epoxidase), respectively. Each plot spans a 3-megabase (Mb) window centered on the target locus, with each dot representing a single nucleotide polymorphism (SNP) in the GWAS or an expressed gene in the TWAS. Co-localization of GWAS and TWAS signals identifies candidate genes that contribute to both genetic and transcriptional variations in metabolite levels.

A total of 301 884 SNPs were obtained from sequencing the *C. chinense* accessions. After filtering for a minor allele frequency (MAF) of 0.05% and a call rate of 70%, 43 081 SNPs were mapped across the 12 pepper chromosomes (Fig. S2 and Table S5). Population stratification based on these filtered SNPs revealed eigen values EV1 = 10.77 and EV2 = 19.63, which were used as parameters for the association studies (Fig. S3). Further, a GWAS analysis was conducted, integrating the metabolome with the *C. chinense* genome to explore the genetic relationships with capsaicinoids, carotenoids, and flavor compounds. The analysis employed an additive model for each metabolite, utilizing correlation and trend tests, linear regression as statistical parameters, and a FDR correction. Manhattan plots for each metabolite were generated to visualize and identify potential genes associated with the traits, with a significance threshold of *P* value ≥3.0 (Fig. 1). Additional Manhattan plots for selected SNP markers for all metabolites are presented in supplementary Figs S4–S6.

### Functional links between mGWAS variants and TWAS-associated genes

To elucidate the genetic architecture underlying complex metabolic traits in *C. chinense*, we employed a combined approach integrating genome-wide association studies (GWAS) and transcriptome-wide association studies (TWAS). GWAS enabled the identification of single-nucleotide polymorphisms (SNPs) that are significantly associated with variation in capsaicinoid, carotenoid, and flavor volatile content. In contrast, TWAS facilitated the discovery of gene expression patterns that correlate with these phenotypic traits. This integrative strategy enabled us to identify high-confidence candidate genes with both structural and regulatory roles, thereby providing a comprehensive understanding of the molecular networks governing trait diversity in pepper. Across all traits examined, we detected a total of 507 SNPs associated with capsaicinoid compounds, including 233 quantitative trait nucleotides (QTNs) associated with capsaicin and 274 with dihydrocapsaicin (DHC). In parallel, 304 SNPs were found to be significantly associated with carotenoid profiles, including 126 for α-carotene, 57 for β-carotene, 48 for capsanthin, and 73 for zeaxanthin. The most extensive number of associations was observed for volatile flavor compounds, with 1176 SNPs identified, including 139 for 2-hexanal, 643 for 4-methylpentyl 2-methylbutanoate, 247 for 4-methylpentyl 3-methylbutanoate, and 148 for 4-methylpentyl 4-methylpentanoate (Table S6).

**Figure 2:**
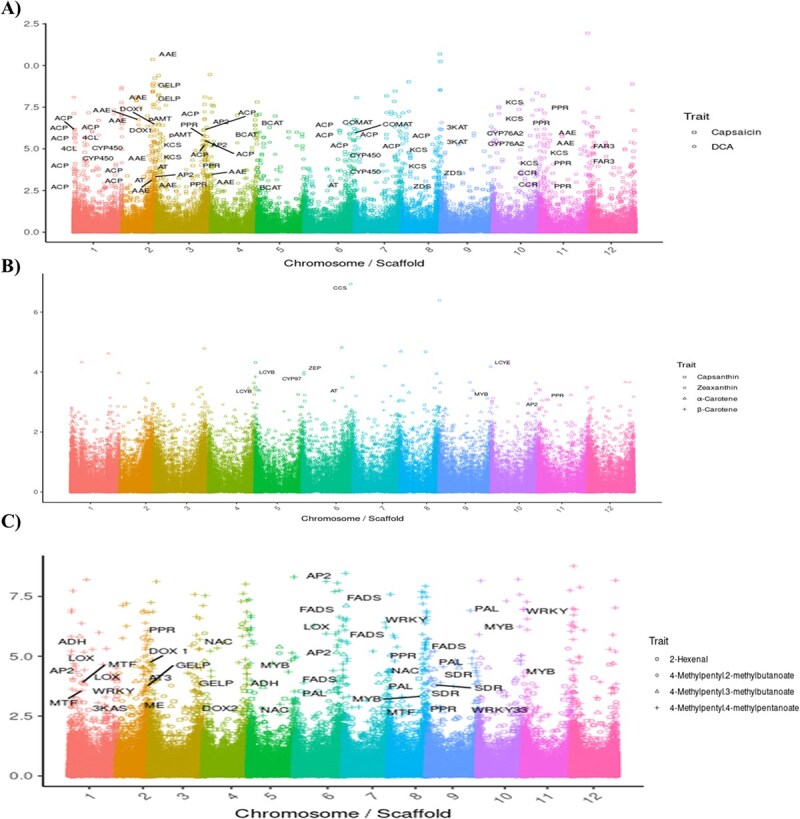
Manhattan plots of transcriptome-wide association studies (TWAS) for three representative metabolites in *C. chinense* fruit: (A) capsaicin, (B) α-carotene, and (C) 4-methylpentyl 4-methylpentanoate. Each point (shape) represents a gene transcript, plotted according to its genomic position and association significance. Key biosynthetic pathway genes and transcriptional regulators identified as significantly associated with metabolite variation are highlighted in the plots. These results illustrate the transcriptional control underlying the biosynthesis of pungency, pigmentation, and flavor volatiles in pepper.

A comparative analysis of GWAS and TWAS results for capsaicinoid content revealed 57 overlapping associations for capsaicin and 59 for DHC, highlighting robust convergence between genetic variants and transcriptomic regulation. GWAS results showed strong associations with key structural genes involved in the capsaicin biosynthetic pathway. These included multiple acyltransferases (AT), acyl carrier proteins (*ACP*), and acyl-activating enzymes (*AAE*), as well as enzymes such as amino acid transferase, branched-chain amino acid aminotransferase (*BCAT*), cinnamoyl-CoA reductase (*CCR*), caffeic acid 3-O-methyltransferase (*COMT*), 4-coumarate-CoA ligase (*4CL*), 3-ketoacyl-CoA synthase (*3KAS*), 3-ketoacyl-CoA thiolase (*3KAT*), and alpha-dioxygenase (*DOX*), all previously implicated in capsaicinoid biosynthesis pathway (Fig. 2 and Fig. S7). The TWAS analysis not only confirmed several of these candidate genes but also uncovered novel associations with transcripts linked to fatty acid metabolism and carotenoid biosynthesis, which are precursors to capsaicinoids. Genes, such as 3-hydroxyisobutyryl-CoA hydrolase 1 (CHY1), 3-ketoacyl-CoA synthases (*KCS*), long-chain alcohol oxidase, glycerol-3-phosphate 2-O-acyltransferase 4, glycerol-3-phosphate acyltransferase 5, very-long-chain 3-oxoacyl-CoA reductase (*VLC*), cytochrome P450s (*CYP450*), fatty acyl-CoA reductase (*FAR3*), and zeta-carotene desaturase (*ZDS*), were identified. In addition, regulatory genes, including multiple AP2-like transcription factors and pentatricopeptide repeat-containing proteins (PPRs), were highlighted, suggesting complex regulation of capsaicinoid biosynthesis at the transcriptional level. These associations were widely distributed across the genome, with prominent loci observed on chromosomes 2, 3, 4, 6, 8, and 11. Notable examples include the *PUN1* locus on chromosome 2, which encodes *AT* genes (*CC.CCv1.2.scaffold266.71* and *CC.CCv1.2.scaffold266.72*) and *AAE* genes (*CC.CCv1.2.scaffold266.114* and *CC.CCv1.2.scaffold266.117*). The *PUN2* region on chromosome 3 was associated with *AMT* (*CC.CCv1.2.scaffold458.4*), while the *BCAT* gene was identified on chromosome 4 (*CC.CCv1.2.scaffold303.50*). Chromosome 6 harbored strong associations with *COMT* (*CC.CCv1.2.scaffold589.76*) and *ACP* (*CC.CCv1.2.scaffold1008.8*). On chromosome 11, loci included *CCR* (CC.CCv1.2.scaffold498.3)c, *AAE* (*CC.CCv1.2.scaffold958.2*), *KCS* (*CC.CCv1.2.scaffold375.8*), and regulatory PPR genes (*CC.CCv1.2.scaffold439.2* and *CC.CCv1.2.scaffold516.15*). Notably, these genomic intervals exhibited similar association patterns for both capsaicin and DHC, reinforcing the hypothesis that the biosynthetic and regulatory mechanisms for these two major capsaicinoids are largely shared (Table S7, Figs 1, 2, and Figs S4, S7).

For carotenoid-related traits, the integration of GWAS and TWAS revealed five shared associations for α-carotene, one for β-carotene, five for capsanthin, and one for zeaxanthin. A major GWAS peak for α-carotene was located at 256.5 Mbp on chromosome 9, corresponding to the lycopene ε-cyclase (*LCYE*) gene, a critical enzyme for α-branch carotenoid biosynthesis. Co-associated transcripts included AP2-like transcription factors. For β-carotene, a peak was detected at 1.8 Mbp on chromosome 5, mapping to lycopene β-cyclase (*LCYB*), which catalyzes the cyclization of lycopene into β-carotene. A capsanthin-associated peak was identified at 239.8 Mbp on chromosome 6, encoding capsanthin/capsorubin synthase (*CCS*), a gene previously characterized for its role in the production of red pigments. Interestingly, LCYB was also significantly associated with capsanthin, suggesting its involvement in multiple carotenoid branches. Additional associations for capsanthin included cytochrome 97B (*C97B*). Zeaxanthin content was linked to zeaxanthin epoxidase (*ZEP*), with a significant GWAS peak near 7 Mbp on chromosome 6. TWAS further revealed that key regulatory genes, such as *AP2* transcription factors and PPRs, were differentially expressed about carotenoid accumulation. All candidate genes associated with carotenoid biosynthesis showed significant differences in expression (FPKM values) between high- and low-content phenotypic lines (Figs 1, 2 and Figs S5, S8, S10), underscoring their functional relevance (Tables S6 and S7) While known biosynthetic genes such as *AMT*, *BCAT*, *4CL*, *COMT*, and *3KAS* featured prominently in these associations, regulatory factors emerged as critical drivers of metabolic variation. Notably, multiple AP2-like transcription factors and Pentatricopeptide Repeats were identified through TWAS as significantly correlated with both carotenoid and volatile compound traits. Their expression patterns showed strong correlation with key biosynthetic enzymes, suggesting that AP2s exert hierarchical control over downstream metabolic pathways. The co-localization of AP2 and PPR genes with key biosynthetic loci, such as near LCYE (chromosome 9), further supports their role in the transcriptional regulation of carotenoid biosynthesis (Fig. 2). AP2s were also found in proximity to major volatile-associated SNP clusters, including those linked to esters and aldehydes. These findings reveal AP2s as regulatory integrators of diverse metabolic pathways in pepper fruits.

Flavor volatiles also showed strong evidence of genetic and transcriptional control. Eight shared associations were observed for 2-hexanal, along with 26 for 4-methylpentyl 2-methylbutanoate, 15 for 4-methylpentyl 3-methylbutanoate, and 35 for 4-methylpentyl 4-methylpentanoate. GWAS analysis identified a strong signal for 2-hexanal at 257.8 Mbp on chromosome 3, mapping to dioxygenase 2 (*DOX2*), and another at 177.5 Mbp on chromosome 8 near a PPR gene. TWAS highlighted alcohol dehydrogenase (*ADH*) as a key transcript associated with 2-hexanal, indicating its role in aldehyde metabolism. For 4-methylpentyl 2-methylbutanoate, significant peaks were detected at 190.1 Mbp on chromosome 8 (*CHY1*) and 33.1 Mbp on chromosome 9 (short-chain dehydrogenase, *SDH*). TWAS further identified several candidate genes, including multiple methyltransferases (*MTFs*) and PPRs, which are likely involved in the enzymatic and regulatory modification of ester volatiles. Similarly, 4-methylpentyl 3-methylbutanoate showed strong associations at 60 Mbp on chromosome 1 (N-methyltransferase), 153.8 Mbp on chromosome 2 (*GDSL* esterase/lipase), 129.8 Mbp on chromosome 3 (UfaA1-like methyltransferase), and 227.9 Mbp on chromosome 7 (1-acyl-sn-glycerol-3-phosphate acyltransferase). TWAS corroborated these findings by identifying transcripts for methyltransferase 1, *SDH*, and PPRs, reinforcing their roles in flavor compound biosynthesis. For 4-methylpentyl 4-methylpentanoate, we identified significant GWAS peaks at 227.1 Mbp on chromosome 6 and 2.1 Mbp on chromosome 9, corresponding to multiple omega-6 fatty acid desaturase (*FADS2*) genes. Another significant locus was found at 184.3 Mbp on chromosome 8, encoding a germin-like protein subfamily 1 member. Additionally, MYB transcription factors located at 232.3 Mbp on chromosome 4 were associated with this compound, indicating a broader regulatory influence. Several other genes were implicated across multiple flavor volatiles, including linoleate 13S-lipoxygenase (*LOX*), polyphenol oxidase (*PPO*), dioxygenase 1 (*DOX1*), esterases, and phenylalanine ammonia-lyase (*PAL*). Regulatory transcription factors such as AP2, NAC, PPRs, and MYBs were also recurrently associated, suggesting that flavor biosynthesis in pepper is under complex transcriptional regulation (Fig. 2 and Figs S6–S9). Together, these findings demonstrate the power of combining GWAS and TWAS to reveal both structural and regulatory genes controlling essential metabolic traits in *C. chinense*. The identification of overlapping loci across methods reinforces the robustness of candidate gene selection and provides a foundation for further functional validation.

**Figure 3:**
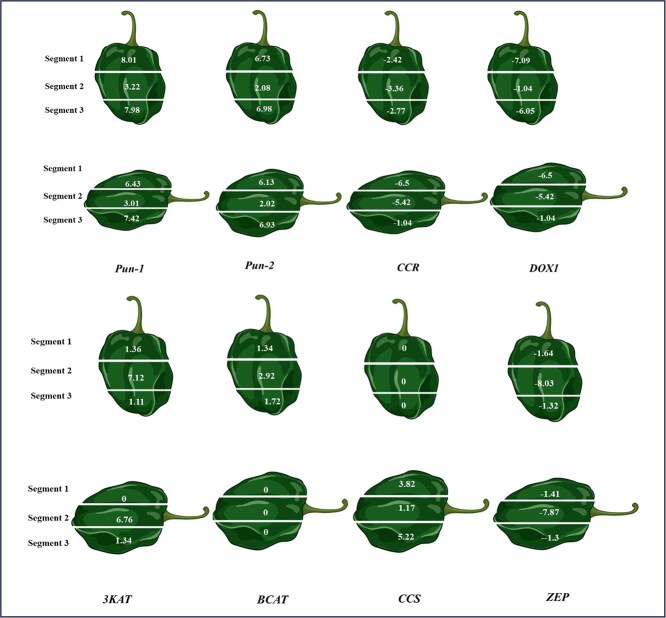
Expression patterns of key candidate genes identified through metabolite genome-wide association studies (mGWAS) for capsaicinoids, carotenoids, and flavor compounds in two contrasting *Capsicum chinense* accessions (PI 656271 and PI 660973). The figure highlights selected genes exhibiting the most significant expression variability across vertical and horizontal sections of 20-day post-anthesis (20-dpa) fruits. These spatial expression differences provide insight into the tissue-specific regulation of metabolic pathways that contribute to pungency, pigmentation, and aroma profiles in pepper fruits.

### Segmental transcriptomic profiling validates and enhances GWAS- and TWAS-inferred candidate gene expression in *C. chinense*

Metabolite profiling of contrasting *C. chinense* accessions—PI 656271 (Costa Rica) and PI 660973 (Colombia) revealed distinct biochemical differences in fruit composition and developmental characteristics. PI 656271 exhibited significantly higher accumulation of capsaicinoids, including capsaicin (4.09 mg/g) and dihydrocapsaicin (1.32 mg/g), compared to PI 660973, which contained 0.038 mg/g and 0.001 mg/g, respectively. Similarly, PI 656271 showed elevated levels of carotenoids, such as α-carotene (4.21 μg/g), β-carotene (5.85 μg/g), and capsanthin (4.13 μg/g). In contrast, PI 660973 had relatively higher levels of certain volatile flavor compounds, notably 4-methylpentyl 3-methylbutanoate (6.20 ng/g). These metabolite contrasts provided a valuable framework for identifying transcriptional patterns associated with trait variation. To validate and expand upon candidate loci identified by GWAS and TWAS analyses, we conducted a segmental RNA-seq-based transcriptomic analysis using fruit tissue sections sampled in both vertical and horizontal orientations, with three segments per orientation and three biological replicates per sample. This spatially resolved transcriptomic approach enabled us to explore metabolic gene expression across specific anatomical zones of the fruit. Sequencing yielded approximately 861 million raw reads, with 766 million high-quality reads successfully mapped to the *C. chinense* reference genome (Table S8). Differential expression analysis using PI 660973 as the reference line identified 7034 differentially expressed genes (DEGs) among the fruit sections (Table S9). Differential gene expression of other genes related to the pathway was mentioned in Table S9 and Fig. 3.

### Validation of capsaicinoid, carotenoids and flavor-associated candidate genes

Segmental transcriptomic analysis of vertical and horizontal sections of pepper fruit (20-day post-anthesis) revealed specific regions associated with genes involved in pungency, flavor, and carotenoid biosynthesis. Expression patterns of pungency-related genes indicated that the top (1st) and bottom (3rd) sections exhibited higher expression levels compared to the middle (2nd) section in both vertical and horizontal orientations. Notably, genes such as *Pun-1, Pun-2, CCR,* and *DOX1* showed elevated expression in the first and third sections. Similarly, some genes encoding uncharacterized proteins followed this expression pattern. Conversely, genes, such as *3KAT, BCAT, FAR3,* and glutathione S-transferase (*GST*), exhibited higher expression in the middle section than in the other two. Additionally, other genes, including *AAE, 3KAS, LCAO, CHY1, VLC*, and *AP2*, exhibited differential expression across various fruit sections. *LCYE* showed relatively uniform expression across all fruit sections, whereas *LCYB* exhibited apparent spatial variation. *CCS* demonstrated consistently higher differential expression across all vertical sections of the fruit. In contrast, *ZEP* was more negatively differentially expressed in the middle section compared to the top and bottom, indicating potential spatial regulation of zeaxanthin epoxidation.

### Spatiotemporal transcriptomic expression of contrasting *C. chinense* accessions

**Figure 4:**
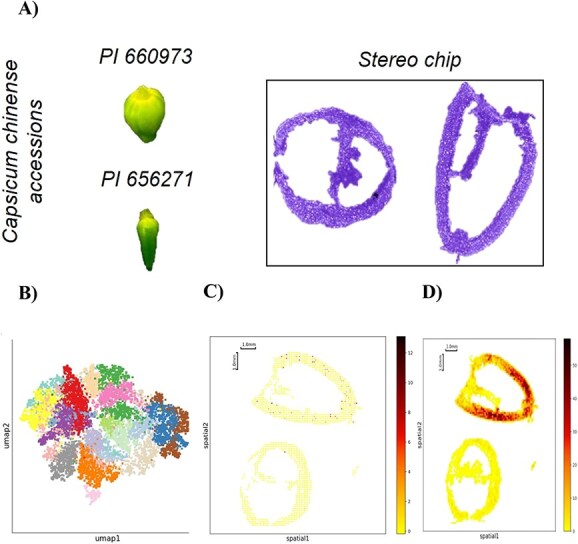
Spatially resolved transcriptomic analysis of two contrasting *C. chinense* accessions, PI 656271 and PI 660973, was performed using Stereo-seq on 5-day post-anthesis (5-dpa) fruits to investigate tissue-specific gene expression patterns. Panel A illustrates the application of spatial transcriptomics to map gene activity within intact fruit tissues. Panel B presents a Uniform Manifold Approximation and Projection (UMAP) plot of spatial transcriptomic spots, where each dot represents an individual spatial location, and colors indicate distinct transcriptional clusters across the fruit sections. Panels C and D display the differential spatial expression of AP2 (APETALA2) transcription factors and PPR (Pentatricopeptide Repeat) genes, respectively, within Modules 1 and 2—gene modules identified as highly spatially correlated and differentially expressed between the two accessions. These spatial expression profiles reveal zone-specific regulatory networks involved in the biosynthesis of capsaicinoids, carotenoids, and volatile metabolites, offering insights into the tissue-level transcriptional control of fruit quality traits in *C. chinense*.

To dissect the spatial regulation of gene expression contributing to metabolite variation in *Capsicum chinense*, we employed high-resolution spatial enhanced omics sequencing (Stereo-seq) in early developmental stage fruits (5 days post-anthesis) of two genetically and biochemically contrasting accessions: PI 656271 and PI 660973. These accessions were previously characterized for differential levels of capsaicinoids, carotenoids, and flavor volatiles. The integration of spatial transcriptomics with GWAS and segmental RNA-seq enabled us to map gene expression dynamics *in situ*, providing insight into cell-type and tissue-specific regulation of key metabolic traits. Stereo-seq analysis generated a total of 78 550 383 uniquely mapped reads, aligned to transcripts from at least one gene in the *C. chinense* reference genome. A total of 21 870 genes were detected under tissue coverage, with an average of 582 unique transcripts (Tables S10 and S11). Unsupervised spot clustering using spatial gene expression data identified 29 transcriptionally distinct clusters, which were visualized through Uniform Manifold Approximation and Projection (UMAP) (Fig. 4B). UMAP embedding revealed well-separated tissue domains in both accessions, with notable differences in the spatial distribution of transcriptional clusters (Fig. 6C). To identify spatially informative genes with significant local autocorrelation, we applied the Hotspot tool, which evaluates pairwise gene–gene expression correlation across spatial coordinates (Fig. S11). This analysis grouped spatially variable genes into six correlation modules (Fig. S8). Among them, Modules 1 and 2 exhibited the most striking expression contrasts between PI 656271 and PI 660973 (Fig. 4). Module 1, comprising 219 genes, included transcripts, such as aspartyl aminopeptidase, importin-5-like isoform, and photosystem II 10 kDa polypeptide, while Module 2 contained 30 genes, including ornithine decarboxylase, serine carboxypeptidase, and pentatricopeptide repeat-containing proteins (PPRs). These genes displayed differential spatial expression across tissue regions, potentially reflecting diverse developmental or metabolic states in fruit anatomy. Crucially, a comparative analysis of GWAS, segmental RNA-seq, and Stereo-seq data revealed 74 spatially correlated genes that overlapped with metabolite-associated loci (Table S11). These include transcription factors and structural genes involved in capsaicinoid (e.g. *3-ketoacyl-CoA synthase 6*, *acyl carrier protein*), carotenoid (e.g. *lycopene β-cyclase*, *ethylene-responsive transcription factor*), and flavor biosynthesis (e.g. SWEET10, ethylene signaling components). Among the key regulatory elements, AP2 transcription factors emerged as important spatial regulators, particularly in carotenoid- and volatile-enriched zones. Several AP2-like genes showed strong expression localized to the exocarp and placental tissue, regions that coincided with high carotenoid content and ethylene-related activity. The spatial co-expression of AP2-like transcription factors with biosynthetic genes, such as capsanthin/capsorubin synthase, lycopene β-cyclase, and zeaxanthin epoxidase, suggests that AP2s may play a pivotal role in orchestrating localized gene expression required for pigment accumulation and ripening-related processes. The presence of AP2s in modules with high autocorrelation across spatial domains further supports their function as master regulators of metabolic compartmentalization. Furthermore, the spatial distribution of other transcription factors, such as ethylene-responsive factor, was highly correlated with zones of carotenoid and capsaicinoid biosynthesis, while *3-ketoacyl-CoA synthases* and *acyl carrier proteins* were enriched in regions consistent with pungency-associated metabolite accumulation. Together with AP2s, these regulators form a layered network coordinating developmentally timed and tissue-specific metabolite biosynthesis. This spatiotemporal organization likely underpins the phenotypic divergence observed between PI 656271 and PI 660973, offering mechanistic insights into how transcriptional localization contributes to fruit sensory trait diversity. The inclusion of AP2 transcription factors as spatially enriched and metabolite-associated regulators strongly supports their central role in metabolic programming of *C. chinense* fruit. These findings underscore the power of integrating Stereo-seq with GWAS and segmental transcriptomics to uncover the cell-type–specific and tissue-resolved regulatory logic driving key metabolic traits in pepper. A complete annotation of spatially resolved candidate genes is provided in Supplementary Table S10 and Fig. 4.

### Widely targeted metabolomics and carotenoid profiling of CRISPR/Cas9 knockout plants of candidate genes identified by GWAS

CRISPR/Cas9-mediated knockouts of the *AP2* and *PPR* genes in tomato were performed to validate their functional regulatory roles in fruit metabolism. Widely targeted metabolomics profiling of the mutant fruits revealed extensive changes across diverse metabolic pathways. A total of 2730 unique metabolites were identified, spanning various structural classes, including amino acids, organic acids, lipids, flavonoids, phenolic acids, terpenoids, and plant hormones. Among the edited lines, the *AP2_12.29* mutant exhibited the highest number of differentially accumulated metabolites (1244), followed by *PPR_1.16* (1194), *PPR_1.9* (1172), and *AP2_14.12* (948). Each line demonstrated distinct metabolic shifts compared to the control, with both shared and genotype-specific responses. A core set of 388 metabolites was commonly altered across all knockouts. At the same time, hundreds of unique changes were specific to each line, underscoring the differentiated roles of *AP2* and *PPR* genes in metabolic regulation.

**Figure 5:**
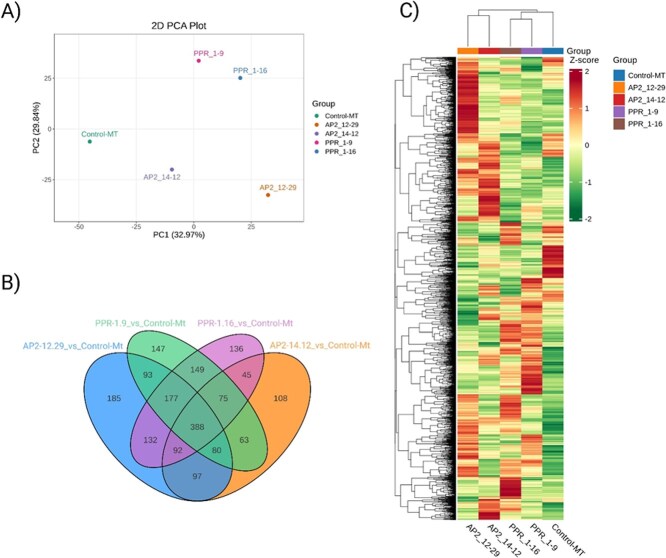
Metabolic profiling of CRISPR/Cas9-edited tomato lines targeting *AP2* (APETALA2) and *PPR* (Pentatricopeptide Repeat) genes reveals distinct alterations in metabolite accumulation. Panel A shows a Principal Component Analysis (PCA), where knockout lines are separated from the wild-type control (Micro-Tom), indicating genotype-specific metabolic shifts. Panel B presents a Venn diagram illustrating the number of unique and shared differentially accumulated metabolites (defined by a fold change ≥2 or ≤0.5 and *P* < 0.05) across each knockout compared to the control, emphasizing both common and gene-specific metabolic effects. Panel C displays a hierarchical clustering heatmap of Z-score–normalized metabolite abundance, which further highlights the divergent metabolic profiles among the *AP2* and *PPR* knockout lines. These results collectively demonstrate that *AP2* and *PPR* genes play distinct regulatory roles in shaping the metabolic landscape of tomato fruit.

Principal component analysis (PCA) separated the mutants from the control, with *AP2* and *PPR* lines forming distinct clusters, indicating strong genotype-specific metabolomic signatures (Fig. 5A). Hierarchical clustering further supported this divergence, with *AP2_12.29* displaying the most distinct metabolic profile. These trends suggest that both *AP2* and *PPR* transcription factors modulate broad metabolite networks, although *AP2* appears to exert a more substantial effect on secondary metabolism and amino acid-related pathways. For example, in *AP2_12.29*, metabolites such as L-homocysteine, alanine betaine, and kaempferol derivatives were significantly upregulated, pointing to enhanced amino acid metabolism and flavonoid biosynthesis. In contrast, *PPR_1.16* showed elevated levels of carotenoid esters such as canthaxanthin and β-citraurin, while levels of lutein and zeaxanthin esters were reduced, suggesting its involvement in xanthophyll ester remodeling. In *AP2* mutants, increased levels of chlorogenic acid, naringenin chalcone, and ferulic acid indicate a shift toward defense-related phenylpropanoid metabolism. Meanwhile, in *PPR* mutants, alterations in linolenic acid, jasmonic acid precursors, and cytokinins reflect disruptions in hormonal and lipid signaling pathways that may affect fruit development and stress responses. Targeted carotenoid profiling further validated the influence of *AP2* and *PPR* on fruit pigmentation. Sixty-eight carotenoid compounds were quantified, including key carotenes (β-carotene, lycopene, phytoene) and xanthophylls (lutein, zeaxanthin, violaxanthin). Differential carotenoid accumulation was observed across all knockout lines. The *AP2_12.29* line showed markedly higher levels of β-carotene (36.80 μg/g vs. 27.55 μg/g in control), lycopene, and phytofluene, along with reduced levels of (E/Z)-phytoene, suggesting increased metabolic flux toward terminal carotenoids. In *PPR* mutants, consistent upregulation of apocarotenoids, such as β-citraurin and violaxanthin dimyristate was observed, while esterified forms of lutein and zeaxanthin were downregulated, particularly in *PPR_1.16*. The *AP2_14.12* line also displayed a unique increase in violaxanthin-myristate-laurate, supporting the presence of distinct regulatory control in carotenoid esterification (Fig. 5B and C). Collectively, these results demonstrate that *AP2* and *PPR* transcription factors play crucial yet distinct roles in regulating metabolic fluxes related to carotenoid biosynthesis, esterification, and broader metabolic pathways in tomato. *AP2* knockouts primarily promote the accumulation of terminal carotenes such as β-carotene and lycopene, while *PPR* knockouts influence xanthophyll ester profiles and apocarotenoid content. These findings support the strategic targeting of *AP2* and *PPR* genes for metabolic engineering aimed at improving fruit quality traits in tomato and potentially other Solanaceous crops.

## Discussion

The integration of genome-wide association studies (GWAS), transcriptome-wide association studies (TWAS), and segmental and spatial transcriptomics has dramatically advanced our understanding of the genetic and regulatory networks underpinning capsaicinoid, carotenoid, and flavor volatile accumulation in *fruits*. *C. chinense* fruits are renowned for their rich repertoire of secondary metabolites, including capsaicinoids, carotenoids, and a diverse array of volatile and non-volatile compounds, all of which contribute to their nutritional value, sensory attributes, and health benefits [7, 22, 23]. The pronounced variation in these metabolites across genotypes underscores the importance of dissecting the genetic architecture underlying their biosynthesis [9, 24].

Capsaicinoids, the alkaloids responsible for pungency, are synthesized via the convergence of phenylpropanoid and fatty acid pathways, with their accumulation tightly regulated by both structural genes and transcription factors. Recent studies have highlighted the central regulatory role of MYB transcription factors, particularly *CaMYB31*, in orchestrating the expression of capsaicinoid biosynthetic genes [25, 26]. This study shows strong correlations between capsaicinoid levels and genes involved in this pathway. Genes, such as 4CL, CCR, and COMT, as well as BCAT and LCAO, were aligned under the proposed pathway. Another important finding was the identification of multiple *ACPs* and *AAEs* across different chromosomes, highlighting their significance in interactions with other genes. The spatial and temporal expression of *MYB31* and other regulatory factors such as *CaMYB48* and *AP2/ERF* family members is critical for tissue-specific metabolite accumulation, as revealed by high-resolution spatial transcriptomics [27, 28]. Notably, the identification of additional loci such as *Pun2* and *Pun3*, encoding *pAMT* and *MYB31,* respectively, has expanded our understanding of the genetic determinants of pungency beyond the classical *Pun1* locus [26, 29, 30].

Carotenoid biosynthesis in *C. chinense* is similarly complex, involving a network of structural genes (e.g. *phytoene synthase*, *lycopene β-cyclase*, *capsanthin/capsorubin synthase*) and regulatory factors. The *OR* gene and phytoene synthase have been validated as key determinants of chromoplast development and carotenoid precursor biosynthesis. Spatial transcriptomics has revealed that transcriptional regulation by AP2, bZIP, and zinc finger proteins is crucial for fine-tuning carotenoid flux [12, 31, 32]. These findings are consistent with observations in other Solanaceae crops, where *AP2*-like transcription factors coordinate chromoplast differentiation and carotenoid accumulation, often acting as master regulators of both developmental and metabolic transitions [11, 33]. This study identified several key regulatory genes (LCYE, LCYB, CCS, ZEP, and C97B) involved in carotenoid biosynthesis and their interrelationships.

The flavor profile of pepper is shaped by a complex interplay of volatile organic compounds (VOCs), including esters, terpenes, aldehydes, and alcohols, whose biosynthesis is closely linked to fatty acid and terpene metabolic pathways [34, 35]. Integrative metabolomics and transcriptomics have identified key genes, such as *ADH*, *AAT*, and *TPS,* as central to volatile accumulation, with transcription factors from the bHLH, MYB, ARF, and IAA families playing fundamental regulatory roles [1, 7, 21]. Notably, multi-omics studies have revealed that the regulation of both carotenoid and VOC pathways often involves overlapping sets of regulatory elements, including *AP2* and MADS-box transcription factors, highlighting their pleiotropic effects on fruit ripening, aroma development, and metabolite flux [36, 37].

The application of spatial transcriptomics, particularly Stereo-seq and segment-specific analyses, has provided unprecedented resolution in mapping gene expression to discrete fruit tissues and developmental zones [18, 21]. This approach has enabled the identification of 74 genes with consistent spatial expression patterns that overlap with GWAS- and TWAS-derived candidate loci, including biosynthetic genes, regulatory factors, and transporters such as the ABC, SWEET, and PDR families [37, 38]. These findings provide mechanistic insights into how cellular compartmentalization and tissue-specific gene regulation contribute to phenotypic diversity in *C. chinense* fruits. Importantly, the convergence of multi-omics data has highlighted candidate genes with strong allelic effects, such as *BIM2*, *peroxidase 5*, and PPR-containing proteins, which were previously uncharacterized in the context of *C. chinense* flavor or nutritional traits. The identification of ankyrin repeat-containing proteins as recurrent hits across GWAS and transcriptomics further substantiates their likely role in capsaicinoid biosynthesis, possibly through signal transduction or protein–protein interactions within biosynthetic complexes [37, 38]. Furthermore, the discovery of regulatory loci associated with flavor volatile biosynthesis, such as ethylene-responsive factors, short-chain dehydrogenases, methyltransferases, and MADS-box transcription factors, underscores the coordinated regulation of fruit quality traits at the transcriptional level [7, 34].

The frequent observation that TWAS identifies additional positive genes not captured by GWAS in the same populations may be due to the presence of unrepresented markers for GWAS [39]. *AP2* and *PPR* proteins were consistently identified as candidate regulators of capsaicinoid, carotenoid and flavor metabolism across complementary analytical platforms in the current study. To validate the regulatory roles *of AP2 and PPR family members*, CRISPR/Cas9-mediated gene editing was employed in tomato. Widely targeted metabolomics revealed that knockout of *AP2* led to significant increases in β-carotene, lycopene, and other terminal carotenoids, while *PPR* knockouts altered xanthophyll esterification and apocarotenoid profiles. In total, over 2700 metabolites were profiled, with *AP2_12.29* exhibiting the most extensive metabolic reprogramming. Targeted carotenoid quantification confirmed that *AP2* influences flux through the carotenoid pathway, whereas *PPR* affects carotenoid storage forms and ester derivatives. KEGG enrichment analysis further showed that these transcription factors regulate interconnected metabolic pathways, including phenylpropanoid biosynthesis, hormone signaling, and flavonoid metabolism. These functional validations substantiate the central role of *AP2* as a master regulator of carotenoid biosynthesis and *PPR* as a modulator of carotenoid remodeling, corroborating their predicted positions in the metabolite regulatory networks.

Collectively, these advances underscore the power of integrating GWAS, TWAS, and spatial transcriptomics to elucidate the genetic and regulatory basis of key fruit quality traits in *Capsicum*. This multi-layered approach not only validated known regulators but also uncovered numerous novel candidates with potential roles in fruit metabolism and development. The resulting framework lays a robust foundation for future CRISPR-mediated functional validation and marker-assisted breeding of elite pepper varieties tailored for enhanced flavor, nutritional value, and consumer preference [7, 12, 25, 34, 38].

## Conclusion

This study represents a significant advancement in understanding the genetic and regulatory landscape of fruit quality traits in *C. chinense*, including capsaicinoid pungency, carotenoid pigmentation, and the biosynthesis of flavor volatiles. Through the integrative application of GWAS, TWAS, segmental RNA-seq, and high-resolution spatial transcriptomics, we identified both structural and regulatory candidate genes with tissue-specific expression patterns tightly linked to metabolite accumulation. Spatial transcriptomics revealed strong co-localization of AP2-like genes with structural biosynthetic genes in carotenoid- and flavor-rich tissue zones, suggesting a hierarchical regulatory role in coordinating plastid development, ripening, and the biosynthesis of secondary metabolites. Likewise, pentatricopeptide repeat (PPR)-containing proteins emerged as potential regulators of post-transcriptional processing, particularly influencing carotenoid esterification and xanthophyll remodeling. To functionally validate these predictions, we employed CRISPR/Cas9 gene editing in tomato to generate knockout lines for selected *AP2* and *PPR* genes. Widely targeted metabolomics across knockout lines revealed large-scale shifts in metabolite profiles, with *AP2_12.29* displaying the most profound changes—over 1200 metabolites were differentially accumulated, including enhanced levels of amino acids, flavonoids, and terpenoid-related intermediates. Targeted carotenoid profiling confirmed that *AP2* disruption led to a significantly increased accumulation of terminal carotenes, such as β-carotene, lycopene, and phytofluene, indicating an upregulated flux through the MEP-isoprenoid pathway. In contrast, *PPR* mutants exhibited decreased levels of lutein and zeaxanthin esters, along with elevated levels of apocarotenoids, such as β-citraurin and canthaxanthin, suggesting a key role in carotenoid remodeling and storage. KEGG pathway enrichment further revealed that both transcription factors influence phenylpropanoid biosynthesis, hormone signaling, and stress-responsive networks. Taken together, these results validate the central regulatory functions of *AP2* and *PPR* in orchestrating metabolic flux and tissue-specific accumulation of nutritionally and sensorially important compounds in fleshy fruits. This functional evidence corroborates our multi-omics predictions and underscores the importance of spatial and temporal transcriptional control in shaping fruit quality traits. The synergy between GWAS, TWAS, and spatial transcriptomics, reinforced by functional genomics, establishes a powerful model for dissecting complex traits in crop species. These insights pave the way for CRISPR-mediated metabolic engineering and marker-assisted selection strategies to develop *Capsicum* cultivars with enhanced nutritional value, improved sensory properties, and greater consumer appeal.

## Materials and methods

### Plant growth and metabolite profiling

A collection of 244 accessions of *C. chinense* obtained from the USDA-ARS Germplasm Resource Information Network, Plant Genetic Resources Conservation Unit (Griffin, GA) (Table S1), representing a diverse range of geographical origins, was grown under greenhouse conditions using a completely randomized block design with three replications over three seasons. Each replication consisted of five plants per accession. The plants were systematically grown under controlled conditions to ensure uniformity and facilitate accurate data collection. Quantitative analysis of metabolites including carotenoids, flavor volatiles, and capsaicinoids was conducted on fruits from each accession grown in three replications. The concentrations of capsaicinoids were measured in (mg/g), while carotenoids were quantified in (μg/g), while flavor volatiles were measured in nanograms per gram, providing comprehensive insights into the biochemical profiles of the different *C. chinense* accessions. This experimental setup ensured robust data collection and evaluated metabolic variations within and between accessions, essential for understanding the genetic factors influencing metabolite production in habanero peppers.

#### Identification and quantification of capsaicinoids, carotenoids, and VOC

Metabolite content (capsaicinoids and carotenoids) was quantified from pepper flesh samples (100 mg powdered) using methods outlined previously [40]. The volatile organic compounds, including 2-hexenal, 4-methyl ethyl 2-methyl butanoate, 4-methyl ethyl 3-methyl butanoate, and 4-methyl ethyl 4-methyl pentanoate, in pepper were analyzed following the method Zhang [41]. The system (Thermo Fisher Scientific, Waltham, MA, USA) consisted of a Trace 1310 gas chromatograph, a Thermo ISQ QD mass detector, and a Triplus RSH autosampler with ITEX-2 (Tenex TA, 80/100 mesh, CTC Analytics, Zwingen, Switzerland). The concentration of each VOC was expressed as 2-octanone equivalent concentration ng/g DW.

#### Genomic DNA extraction and genotyping by-sequencing

Seedlings of *C. chinense* were collected, and genomic DNA (gDNA) was extracted using the DNeasy Plant Mini Kit (QIAGEN, Germany). The extracted samples were digested with the restriction enzyme ApeKI, a type II restriction endonuclease. Sequencing was performed using the Illumina HiSeq 2500 platform following methods described previously [42]. GBS (Genotyping-by-Sequencing) reads obtained from *C. chinense* genotypes were aligned to the reference genome available at http://peppergenome.snu.ac.kr/ using the Burrows–Wheeler–Aligner (BWA) tool (http://bio-bwa.sourceforge.net/). The aligned GBS reads were processed for Single Nucleotide Polymorphism (SNP) calling using the GB-eaSy tool (https://github.com/dpwickland/GB-easy), which saved the variants in Variant Call Format (VCF) for subsequent association analysis. This sequencing and SNP calling approach enabled comprehensive genomic analysis of *C. chinense*, providing crucial data for association studies to understand genetic variations underlying traits of interest in habanero peppers.

#### GWAS of *C. chinense*

The total 43 081 mapped SNPs with minor allele frequency (MAF) ≥ 0.05 and ≥ 90% call rate were utilized for GWAS to identify alleles responsible for metabolite content in *C. chinense*. Associations were estimated using the MLM model of GAPIT v3.0 in R. Manhattan plots were employed to visualize SNPs with the highest associations and annotate their chromosomal positions [43]. A threshold of -Log P of 3 was considered to identify genome-wide significance. These integrated analyses provided comprehensive insights into the genetic basis of metabolite variation in *C. chinense.*

### TWAS to identify genes associated with pungency and *flavor* compounds

A Transcriptome-Wide Association Study (TWAS) was conducted to identify genes associated with pungency and flavor traits in *Capsicum* by integrating gene expression profiles with phenotypic data. RNA was extracted from 77 fruit samples and processed using standard protocols, including mRNA enrichment, cDNA synthesis, and Illumina-based paired-end sequencing. The quality of RNA and libraries was assessed using the Agilent Bioanalyzer and Qubit Fluorometer, and sequencing libraries were prepared using the NEBNext Ultra II RNA Library Prep Kit. After sequencing on the Illumina NextSeq 500 platform, the data were quality-filtered using FastQC [44] and trimmed using Trimmomatic [45]. High-quality reads were aligned to the pepper reference genome using STAR [46], and expression levels were quantified in FPKM using StringTie. Genes with FPKM ≥0.1 in at least 15 samples were retained, yielding 20 978 transcripts for TWAS. Expression values were transformed to a 0 to 1 scale using a percentile-based normalization approach [44], and association analysis was performed using a general linear model in TASSEL v5.0. Genes were considered significant if located within 1 Mb of trait-associated QTNs identified in previous GWAS, and selected candidates were further subjected to functional annotation and pathway enrichment to explore their roles in metabolic pathways influencing arotenoid, flavor and pungency [39, 44]. This TWAS approach enhances the resolution of candidate gene identification by incorporating gene expression as a mediator between genotype and phenotype.

### Segmental RNAseq of contrasting *C. chinense* accessions

To elucidate the transcriptional landscape of *C. chinense*, we employed RNA sequencing (RNA-seq) to analyze gene expression profiles across three different segments in vertical and horizontal incisions of 20-day post-anthesis (20-dpa) fruits from PI 656271 and PI 660973 accessions, which showed contrasting metabolite content. RNA was extracted from each fruit segment in triplicate. The isolated RNA was assessed for degradation and contamination on 1.2% agarose gels. The quantity of the RNA was measured using the Qubit 3.0 Fluorometer. RNAseq libraries were constructed using equal amounts of RNA (1 μg/μl) and the NEBNext Ultra RNA Library Prep Kit for Illumina (New England Biolabs, Ipswich, MA, USA), following the manufacturer's instructions. The libraries were paired-end sequenced at 30X depth on an Illumina NextSeq 500 system with 150-bp reads [47].

### Stereo-seq spatial Transcriptomics of contrasting *C. chinense* accessions

To generate spatially resolved transcriptomic profiles, 5-day post-anthesis (5-dpa) fruits from two contrasting *Capsicum chinense* accessions, PI 656271 and PI 660973, were harvested and processed for Stereo-seq analysis. Fruits were embedded in pre-chilled optimal cutting temperature (OCT) compound (SAKURA) and flash-frozen on dry ice. OCT-embedded tissue blocks were equilibrated at −20°C for 20 min and sectioned at a thickness of 10 μm using a cryostat (Leica CM1950) for both spatial transcriptome sequencing and histological imaging. Stereo-seq transcriptomics was conducted using the BGI Stereo-seq kit (catalog 211ST114, STOmics), following the manufacturer’s instructions with minor modifications [48]. Prior to sample mounting, Stereo-seq chips were coated with 100 μl of 0.01% poly-l-lysine (Sigma) and incubated at room temperature for 10 min. The coating solution was then removed, and chips were washed three times with 100 μl of nuclease-free water. Subsequently, 100 μl of 0.01% Fluorescent Brightener 28 (Sigma) prepared in 0.1× SSC containing 5% RNase inhibitor was applied and incubated for 3 min in the dark at room temperature. The chips were then washed once with 0.1× SSC containing 5% RNase inhibitor, followed by tissue imaging using DAPI and FITC fluorescent channels to visualize nuclei and ssDNA. Tissue sections were adhered to the prepared chip surfaces and fixed with chilled methanol. For tissue permeabilization and RNA capture, chips were incubated with 0.1% proprietary PR enzyme in 0.01 M HCl buffer (pH 2.0) for 17 min at 37°C. This step enabled the hybridization of polyadenylated RNAs to the poly-T oligonucleotides embedded in the chip. Reverse transcription was performed overnight at 37°C in a humidified chamber. Following cDNA synthesis, tissue remnants were removed, and spatially barcoded cDNA was released using cDNA Release Buffer for 3 h at room temperature. The recovered cDNA was used for spatial library preparation following the Stereo-seq Library Preparation Kit (BGI, catalog 111KL114) protocol. Final libraries were sequenced on the DNBSEQ-T5 platform at the STOmics facility (BGI, China). Raw spatial transcriptomic data were aligned to the *C. chinense* reference genome, yielding a total of 78 550 383 aligned reads across both accessions. Transcripts from 21 870 genes were detected within tissue-covered areas, with an average of 582 unique transcripts and genes detected per bin200. Unsupervised spot clustering was performed using the Stereo-seq pipeline to identify expression-based domains, resulting in the identification of 29 discrete transcriptional clusters. The spatial clusters were visualized using Uniform Manifold Approximation and Projection (UMAP) to assess transcriptomic heterogeneity across tissue sections. To further explore spatially informative gene modules, we applied the Hotspot algorithm to identify genes with significant local autocorrelation across spatial coordinates. Informative genes were grouped into six co-expression modules based on local pairwise correlation. Modules 1 and 2 were prioritized for downstream analysis due to their pronounced spatial expression differences between PI 656271 and PI 660973. A total of 74 spatially correlated genes overlapped with candidate genes identified in prior GWAS and segmental RNA-seq analyses (Tables S11). Gene ontology enrichment analysis for module-specific genes was performed using the ClusterProfiler package in R [49], with significant GO terms visualized via dot plots based on q-value thresholds. Enrichment categories were used to infer potential biological functions associated with spatially patterned gene expression, including those linked to capsaicinoid, carotenoid, and flavor compound biosynthesis.

### CRISPR/Cas9-mediated gene editing in tomato

Candidate genes (*AP2/ERF* and *PPR*) were targeted in tomato (*Solanum lycopersicum* cv. ‘Micro-Tom’) using a CRISPR/Cas9 system. Guide RNAs (gRNAs) were designed using CRISPR-P 2.0 to target conserved exonic regions, ensuring high on-target activity and minimal off-target effects. Synthesized gRNA (gRNAs driven by AtU6 promoter) oligonucleotides were cloned into the pCAMBIA1300 binary vector modified pPBV binary vector harboring a ZmCas9 cassette driven by the CaMV 35S promoter. The constructs were introduced into the *Agrobacterium tumefaciens* strain LBA4404 and used to transform the cotyledonary leaf explants. Regenerated shoots were selected on a medium containing kanamycin (100 mg/L). Elongation and rooting were subsequently carried out on the same selective medium to ensure the development of kanamycin-resistant plants (modified protocol used in this study [50]). Putative T0 plants were screened for T-DNA integration by PCR. Homozygous and biallelic T1 mutants were confirmed by Sanger sequencing and used for subsequent metabolite profiling. Null mutants without off-target were advanced for phenotypic and biochemical analyses (Fig. S12).

### Widely targeted metabolomics profiling

Metabolite profiling was performed by Metware Biotechnology Inc. (Wuhan, China) following standard protocols. Briefly, 50 mg of freeze-dried fruit powder was extracted with 1.2 ml of pre-chilled 70% methanol containing internal standards. Samples were vortexed intermittently for 30 min over six cycles, centrifuged at 12 000 rpm for 3 min at 4°C, and filtered through 0.22 μm membranes. Chromatographic separation was carried out on an Agilent SB-C18 column (1.8 μm, 2.1 × 100 mm) using an ExionLC™ AD system. The mobile phase consisted of 0.1% formic acid in water (solvent A) and acetonitrile (solvent B), with a linear gradient elution. Metabolites were detected on QTRAP® 6500+ and TripleTOF® 6600+ mass spectrometers operating in both positive and negative ESI modes. Identification was based on accurate mass, retention time, and MS/MS spectra compared to an in-house MetWare Database (MWDB). Quantification was conducted in MRM mode using Analyst and MultiQuant software. Quality control measures included imputing missing values as one-fifth of the minimum observed value and retaining metabolites with CV < 0.5 in QC samples. Data reliability was assessed through principal component analysis (PCA), hierarchical clustering analysis (HCA), and Pearson correlation. Differential metabolites were defined using a combination of OPLS-DA (VIP ≥ 1) and univariate analysis (fold change ≥2 or ≤0.5, *P* < 0.05), followed by functional annotation via KEGG pathway enrichment

## Supplementary Material

Web_Material_uhaf243

## Data Availability

The genotyping by sequencing data underlying this article are available in the NCBI database at https://www.ncbi.nlm.nih.gov/sra and can be accessed with the BioProject accession number PRJNA1305095. The raw paired-end of the Segmental RNA-Seq data generated during this study have been deposited in the NCBI Sequence Read Archive (SRA) under BioProject accession number PRJNA1130500.

## References

[1] Akhtar A, Asghar W, Khalid N. Phytochemical constituents and biological properties of domesticated capsicum species: a review. Bioact Compd Health Dis. 2021;4:201–25

[2] Jimenez-García SN, Garcia-Mier L, Ramirez-Gomez XS. et al. Characterization of the key compounds of bell pepper by spectrophotometry and gas chromatography on the effects of induced stress on the concentration of secondary metabolite. Molecules. 2023;28:3830 37175241 10.3390/molecules28093830 PMC10180469

[3] Moses M, Umaharan P. Genetic structure and phylogenetic relationships of Capsicum chinense. J Am Soc Hortic Sci. 2012;137:250–62

[4] Villa-Rivera MG, Ochoa-Alejo N. Chili pepper carotenoids: nutraceutical properties and mechanisms of action. Molecules. 2020;25:5573 33260997 10.3390/molecules25235573 PMC7729576

[5] Moreira AFP, Ruas PM, de Fátima RC. et al. Genetic diversity, population structure and genetic parameters of fruit traits in Capsicum chinense. Sci Hortic. 2018;236:1–9

[6] Zhang J, Wang C, Wang J. et al. Comprehensive fruit quality assessment and identification of aroma-active compounds in green pepper (*Capsicum annuum* L.). Front Nutr. 2023;9:1027605 36704799 10.3389/fnut.2022.1027605 PMC9871545

[7] Joshi DD, Somkuwar BG, Kharkwal H. et al. Aroma based varieties of *Capsicum chinense* Jacq., geographical distribution and scope for expansion of the species. J Appl Res Med Aromatic Plants. 2022;29:100379

[8] Chevalier W, Moussa S-A, Medeiros Netto Ottoni M. et al. Multisite evaluation of phenotypic plasticity for specialized metabolites, some involved in carrot quality and disease resistance. PLoS One. 2021;16:e0249613 33798246 10.1371/journal.pone.0249613 PMC8018645

[9] Wahyuni Y, Ballester A-R, Sudarmonowati E. et al. Secondary metabolites of capsicum species and their importance in the human diet. J Nat Prod. 2013;76:783–93 23477482 10.1021/np300898z

[10] Zhang W, Wu D, Zhang L. et al. Identification and expression analysis of capsaicin biosynthesis pathway genes at genome level in *Capsicum chinense*. Biotechnol Biotechnol Equip. 2022;36:232–44

[11] Yuan P, Umer MJ, He N. et al. Transcriptome regulation of carotenoids in five flesh-colored watermelons (*Citrullus lanatus*). BMC Plant Biol. 2021;21:203 33910512 10.1186/s12870-021-02965-z PMC8082968

[12] Zhou X, Liu Z. Unlocking plant metabolic diversity: a (pan)-genomic view. Plant Commun. 2022;3:100300 35529944 10.1016/j.xplc.2022.100300 PMC9073316

[13] Rahimi Y, Khahani B, Jamali A. et al. Genome-wide association study to identify genomic loci associated with early vigor in bread wheat under simulated water deficit complemented with quantitative trait loci meta-analysis. G3 (Bethesda). 2023;13:jkac320 36458966 10.1093/g3journal/jkac320 PMC10248217

[14] Zuo J, Wang Y, Zhu B. et al. Network analysis of noncoding RNAs in pepper provides insights into fruit ripening control. Sci Rep. 2019;9:8734 31217463 10.1038/s41598-019-45427-1 PMC6584694

[15] Rothan C, Diouf I, Causse M. Trait discovery and editing in tomato. Plant J. 2019;97:73–90 30417464 10.1111/tpj.14152

[16] Kremling KA, Diepenbrock CH, Gore MA. et al. Transcriptome-wide association supplements genome-wide association in *Zea mays*. G3 (Bethesda). 2019;9:3023–33 31337639 10.1534/g3.119.400549 PMC6723120

[17] Ming L, Fu D, Wu Z. et al. Transcriptome-wide association analyses reveal the impact of regulatory variants on rice panicle architecture and causal gene regulatory networks. Nat Commun. 2023;14:7501 37980346 10.1038/s41467-023-43077-6 PMC10657423

[18] Giacomello S, Salmén F, Terebieniec BK. et al. Spatially resolved transcriptome profiling in model plant species. Nat Plants. 2017;3:1–11 10.1038/nplants.2017.61 28481330

[19] Liu Z, Yang B, Zhang T. et al. Full-length transcriptome sequencing of pepper fruit during development and construction of a transcript variation database. Hortic Res. 2024;11:uhae198 39257544 10.1093/hr/uhae198 PMC11387007

[20] Lv K, Liu N, Niu Y. et al. Spatial transcriptome analysis reveals de novo regeneration of poplar roots. Hortic Res. 2024;11:uhae237 39512783 10.1093/hr/uhae237 PMC11540759

[21] Gurazada SGR, Cox KL Jr, Czymmek KJ. et al. Space: the final frontier—achieving single-cell, spatially resolved transcriptomics in plants. Emerg Topics Life Sci. 2021;5:179–88 10.1042/ETLS20200274 33522561

[22] Azlan A, Sultana S, Huei CS. et al. Antioxidant, anti-obesity, nutritional and other beneficial effects of different chili pepper: a review. Molecules. 2022;27:898 35164163 10.3390/molecules27030898 PMC8839052

[23] Olatunji TL, Afolayan AJ. The suitability of chili pepper (*Capsicum annuum* L.) for alleviating human micronutrient dietary deficiencies: a review. Food Sci Nutr. 2018;6:2239–51 30510724 10.1002/fsn3.790 PMC6261225

[24] Nimmakayala P, Lopez-Ortiz C, Shahi B. et al. Exploration into natural variation for genes associated with fruit shape and size among *Capsicum chinense* collections. Genomics. 2021;113:3002–14 34229041 10.1016/j.ygeno.2021.06.041

[25] Arce-Rodríguez ML, Ochoa-Alejo N. An R2R3-MYB transcription factor regulates capsaicinoid biosynthesis. Plant Physiol. 2017;174:1359–70 28483879 10.1104/pp.17.00506 PMC5490919

[26] Zhu Z, Sun B, Cai W. et al. Natural variations in the MYB transcription factor MYB31 determine the evolution of extremely pungent peppers. New Phytol. 2019;223:922–38 31087356 10.1111/nph.15853

[27] Licausi F, Ohme-Takagi M, Perata P. APETALA 2/ethylene responsive factor (AP 2/ERF) transcription factors: mediators of stress responses and developmental programs. New Phytol. 2013;199:639–49 24010138 10.1111/nph.12291

[28] Sun T, Li L. Toward the ‘golden’ era: the status in uncovering the regulatory control of carotenoid accumulation in plants. Plant Sci. 2020;290:110331 31779888 10.1016/j.plantsci.2019.110331

[29] Koeda S, Sato K, Tomi K. et al. Analysis of non-pungency, aroma, and origin of a *Capsicum chinense* cultivar from a Caribbean island. J Japan Soc Hortic Sci. 2014;83:244–51

[30] Stewart C Jr, Kang BC, Liu K. et al. The Pun1 gene for pungency in pepper encodes a putative acyltransferase. Plant J. 2005;42:675–88 15918882 10.1111/j.1365-313X.2005.02410.x

[31] He X, Liu K, Wu Y. et al. A transcriptional cascade mediated by two APETALA2 family members orchestrates carotenoid biosynthesis in tomato. J Integr Plant Biol. 2024;66:1227–41 38546046 10.1111/jipb.13650

[32] Li Q-H, Yang S-P, Yu Y-N. et al. Comprehensive transcriptome-based characterization of differentially expressed genes involved in carotenoid biosynthesis of different ripening stages of capsicum. Sci Hortic. 2021;288:110311

[33] Stanley L, Yuan Y-W. Transcriptional regulation of carotenoid biosynthesis in plants: so many regulators, so little consensus. Front Plant Sci. 2019;10:1017 31447877 10.3389/fpls.2019.01017 PMC6695471

[34] Dong T, Tian Z, Wang S. et al. Identification of key off-flavor compounds during storage of fried pepper (*Zanthoxylum bungeanum* Maxim.) oils by sensory-directed flavor analysis and partial least squares regression (PLSR). J Food Compos Anal. 2024;131:106268

[35] Huang C, Sun P, Yu S. et al. Analysis of volatile aroma components and regulatory genes in different kinds and development stages of pepper fruits based on non-targeted metabolome combined with transcriptome. Int J Mol Sci. 2023;24:7901 37175606 10.3390/ijms24097901 PMC10178352

[36] Fujisawa M, Ito Y. The regulatory mechanism of fruit ripening revealed by analyses of direct targets of the tomato MADS-box transcription factor RIPENING INHIBITOR. Plant Signal Behav. 2013;8:371–86 10.4161/psb.24357 PMC3907458 23518588

[37] Nimmakayala P, Abburi VL, Saminathan T. et al. Genome-wide diversity and association mapping for capsaicinoids and fruit weight in *Capsicum annuum* L. Sci Rep. 2016;6:38081 27901114 10.1038/srep38081 PMC5128918

[38] Liu Y, Lv J, Liu Z. et al. Integrative analysis of metabolome and transcriptome reveals the mechanism of color formation in pepper fruit (*Capsicum annuum* L.). Food Chem. 2020;306:125629 31629298 10.1016/j.foodchem.2019.125629

[39] Li D, Wang Q, Tian Y. et al. TWAS facilitates gene-scale trait genetic dissection through gene expression, structural variations, and alternative splicing in soybean. Plant Commun. 2024;5:101010 38918950 10.1016/j.xplc.2024.101010 PMC11573905

[40] Chebrolu KK, Jayaprakasha G, Jifon J. et al. Production system and storage temperature influence grapefruit vitamin C, limonoids, and carotenoids. J Agric Food Chem. 2012;60:7096–103 22742827 10.1021/jf301681p

[41] Zhang L, Ku K-M. Biomarkers-based classification between green teas and decaffeinated green teas using gas chromatography mass spectrometer coupled with in-tube extraction (ITEX). Food Chem. 2019;271:450–6 30236701 10.1016/j.foodchem.2018.07.137

[42] Elshire RJ, Glaubitz JC, Sun Q. et al. A robust, simple genotyping-by-sequencing (GBS) approach for high diversity species. PLoS One. 2011;6:e19379 21573248 10.1371/journal.pone.0019379 PMC3087801

[43] Wang J, Zhang Z. GAPIT version 3: boosting power and accuracy for genomic association and prediction. Genom Proteom Bioinform. 2021;19:629–40 10.1016/j.gpb.2021.08.005 PMC9121400 34492338

[44] Nawade B, Shim S-H, Chu S-H. et al. Integrative transcriptogenomic analyses reveal the regulatory network underlying rice eating and cooking quality and identify a role for alpha-globulin in modulating starch and sucrose metabolism. Plant Commun. 2025;6:101287 39980198 10.1016/j.xplc.2025.101287 PMC12143143

[45] Bolger AM, Lohse M, Usadel B. Trimmomatic: a flexible trimmer for Illumina sequence data. Bioinformatics. 2014;30:2114–20 24695404 10.1093/bioinformatics/btu170 PMC4103590

[46] Dobin A, Davis CA, Schlesinger F. et al. STAR: ultrafast universal RNA-seq aligner. Bioinformatics. 2013;29:15–21 23104886 10.1093/bioinformatics/bts635 PMC3530905

[47] Natarajan P, Akinmoju TA, Nimmakayala P. et al. Integrated metabolomic and transcriptomic analysis to characterize cutin biosynthesis between low-and high-cutin genotypes of *Capsicum chinense* Jacq. Int J Mol Sci. 2020;21:1397 32092953 10.3390/ijms21041397 PMC7073079

[48] Chen A, Liao S, Cheng M. et al. Spatiotemporal transcriptomic atlas of mouse organogenesis using DNA nanoball-patterned arrays. Cell. 2022;185:1777–1792.e21 e21 35512705 10.1016/j.cell.2022.04.003

[49] Yu G, Wang L-G, Han Y. et al. clusterProfiler: an R package for comparing biological themes among gene clusters. Omics. 2012;16:284–7 22455463 10.1089/omi.2011.0118 PMC3339379

[50] Sun H-J, Uchii S, Watanabe S. et al. A highly efficient transformation protocol for Micro-Tom, a model cultivar for tomato functional genomics. Plant Cell Physiol. 2006;47:426–31 16381658 10.1093/pcp/pci251

