# Supplementary material for: Uncovering the genetic architecture of pungency, carotenoids, and flavor in Capsicum chinense via TWAS-mGWAS integration and spatial transcriptomics
Source: Hortic Res. 2025 Sep 15;12(12):uhaf243. doi: 10.1093/hr/uhaf243 (PMC12701575; doi:10.1093/hr/uhaf243)
Supplement: Web_Material_uhaf243 [file web_material_uhaf243.zip › Supplementary Figures(1).docx]

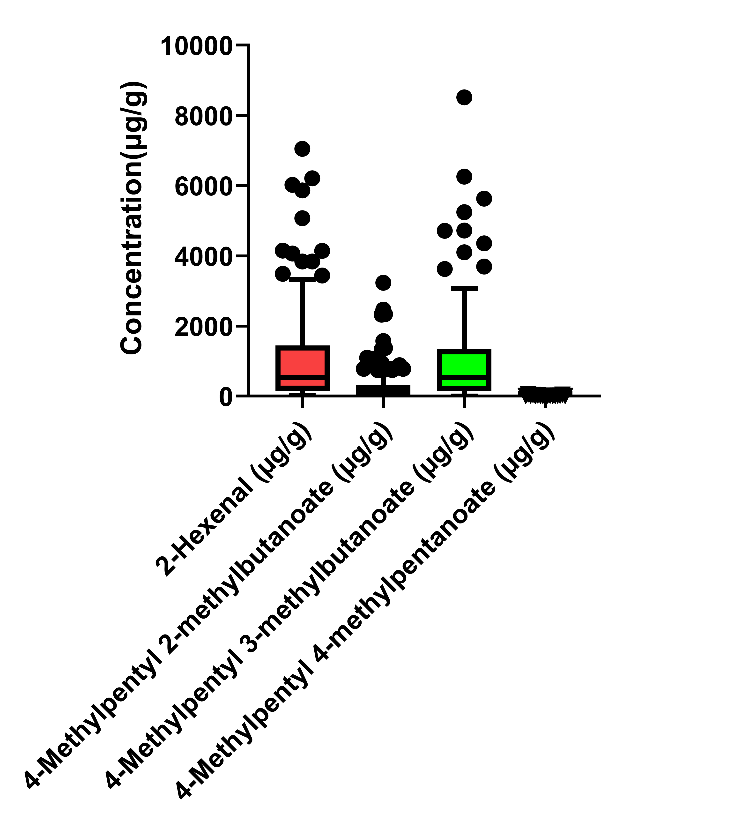

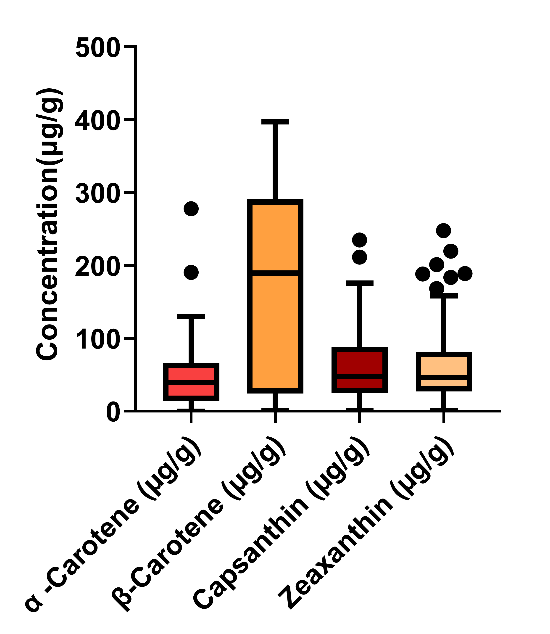

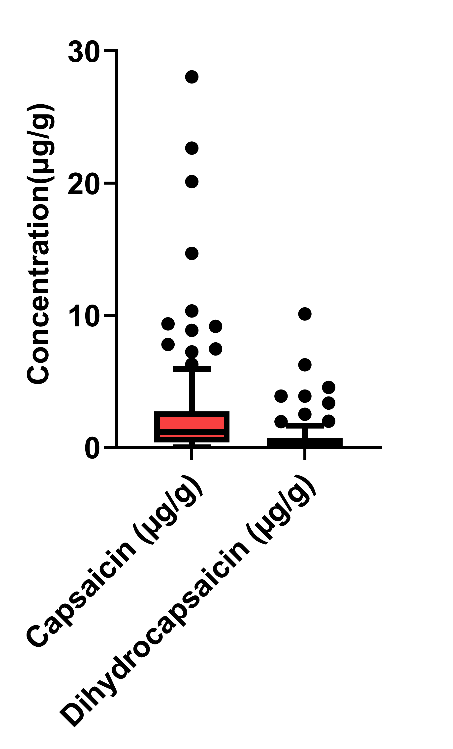


**Figure S1**. Metabolite concentrations of capsaicinoids (A), carotenoids (B), and volatile/non-volatile compounds (C) datasets across *C. chinense* population.

**
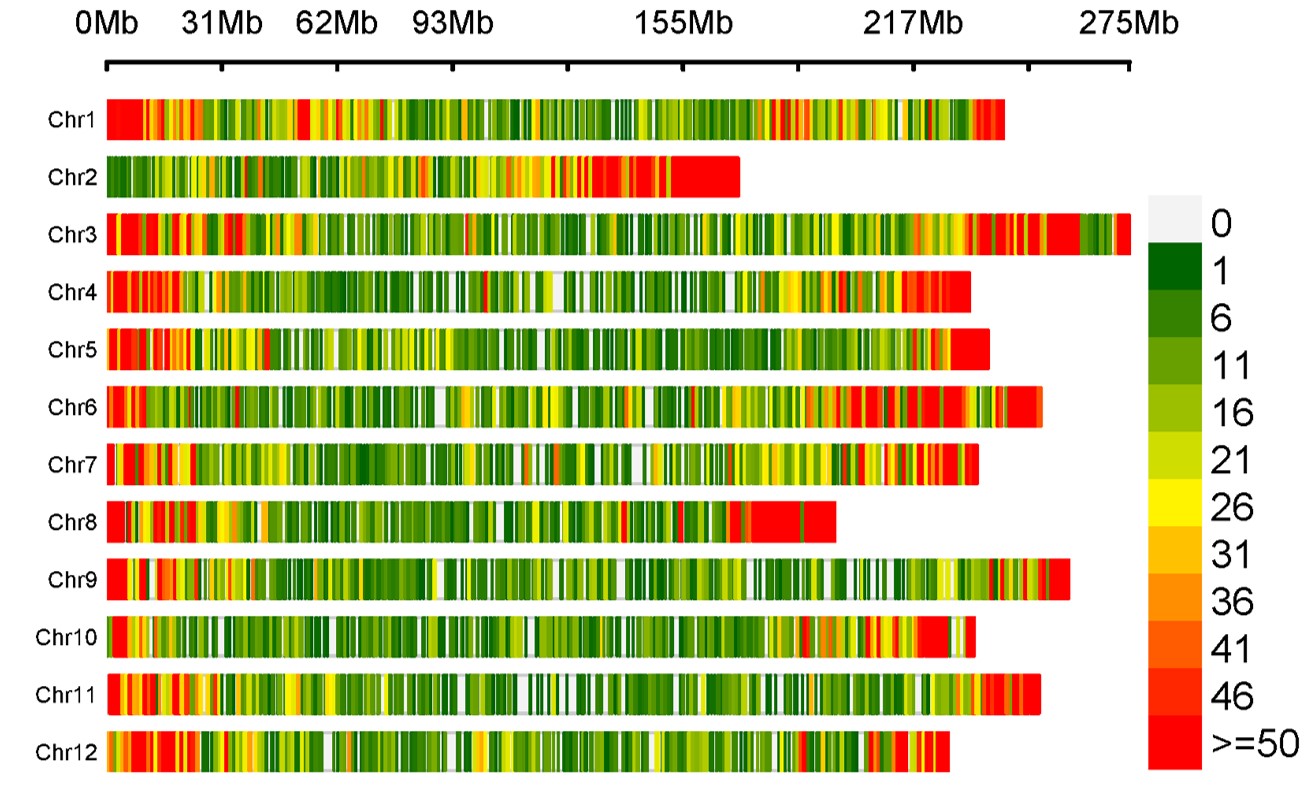
Figure S2**. Chromosome-wise SNP density for *C. chinense*. The number of SNPs is represented within a 1 Mb window size. The horizontal axis shows the chromosome (Chr) length (Mb).

**
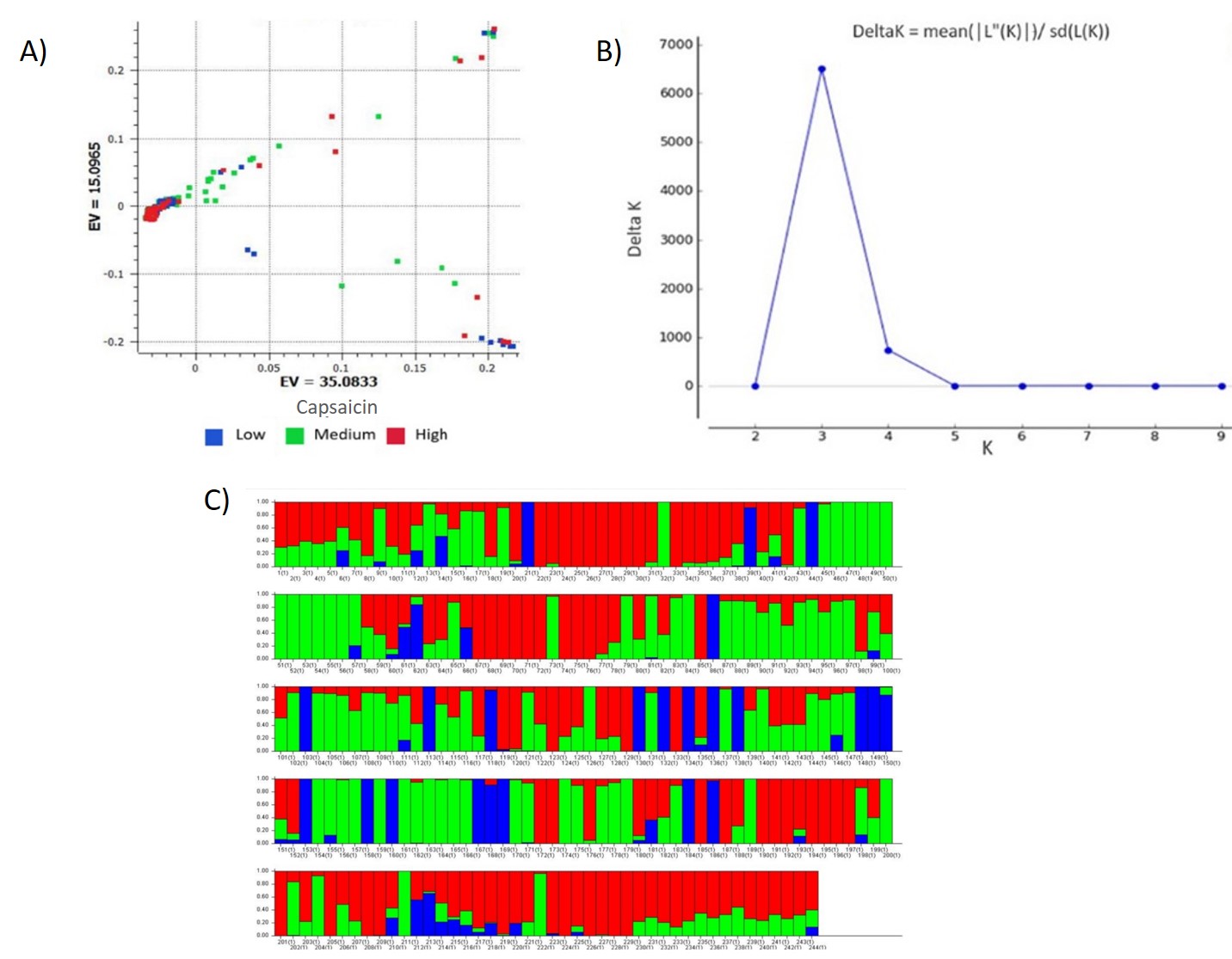
**

**Figure S3**. Principal component analysis and population structure of 244 *C. chinense* accessions were conducted using Structure v.2.3.4 and 43,081 SNP markers. (A) PCA analysis grouped the accessions by capsaicin content. (B) Delta K3 showed the highest peak (based on Delta K distribution), and (C) presents the clustering assignments of the 244 *C. chinense* accessions based on ADMIXTURE analysis for inferred K = 3. Each color represents a different cluster, with the y-axis displaying the estimated percentage membership of each accession in a given cluster.


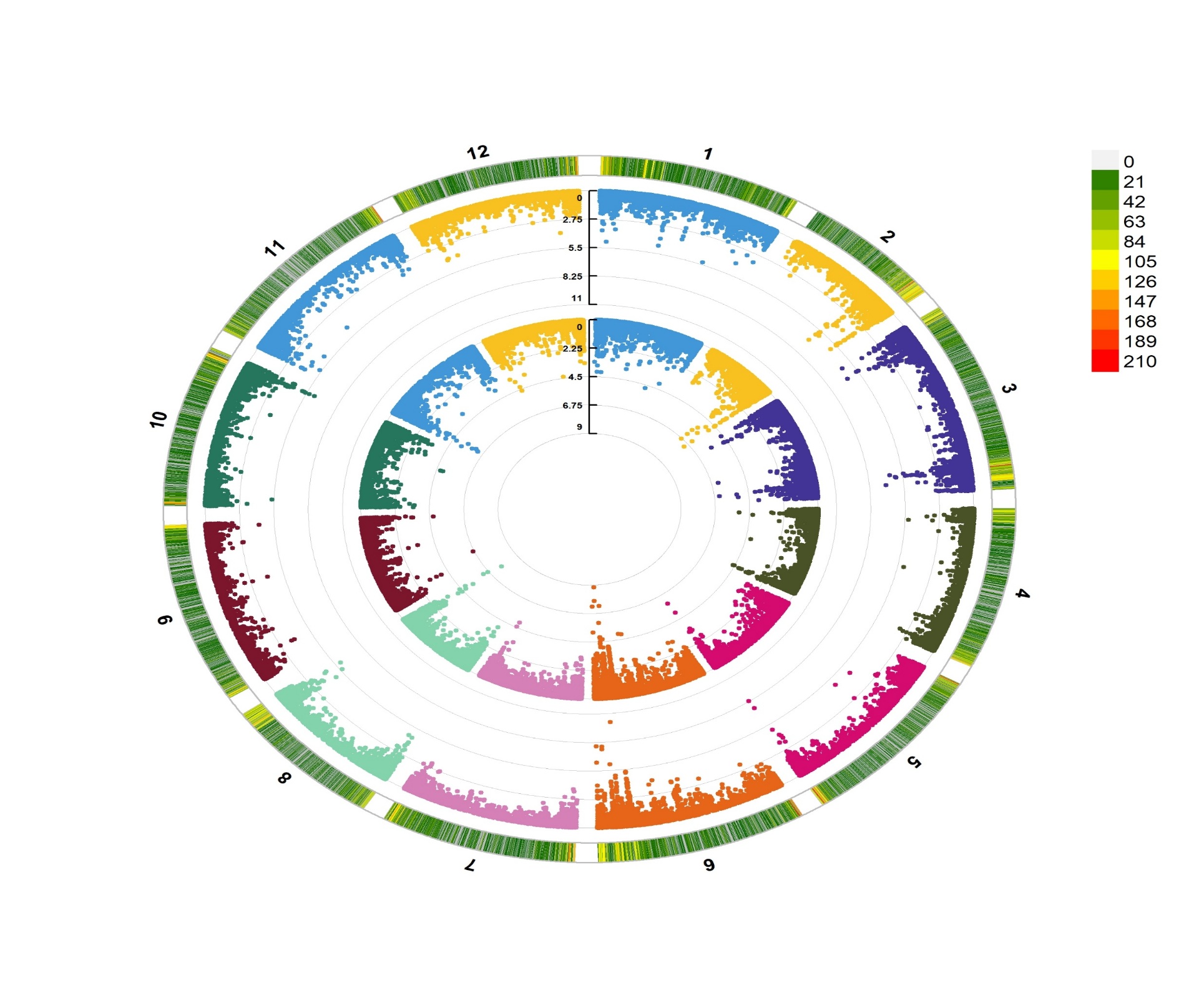


**Figure S4**. Manhattan plot showing SNPs significantly associated with the Capsaicinoids traits in A) Capsaicin (inner most circle) and B) Dihydrocapsaicin (Outer circe) in *C. chinense*. Different colors on the x-axis indicate 12 chromosomes of the pepper genome. Values on the y-axis are P values of -log 10.


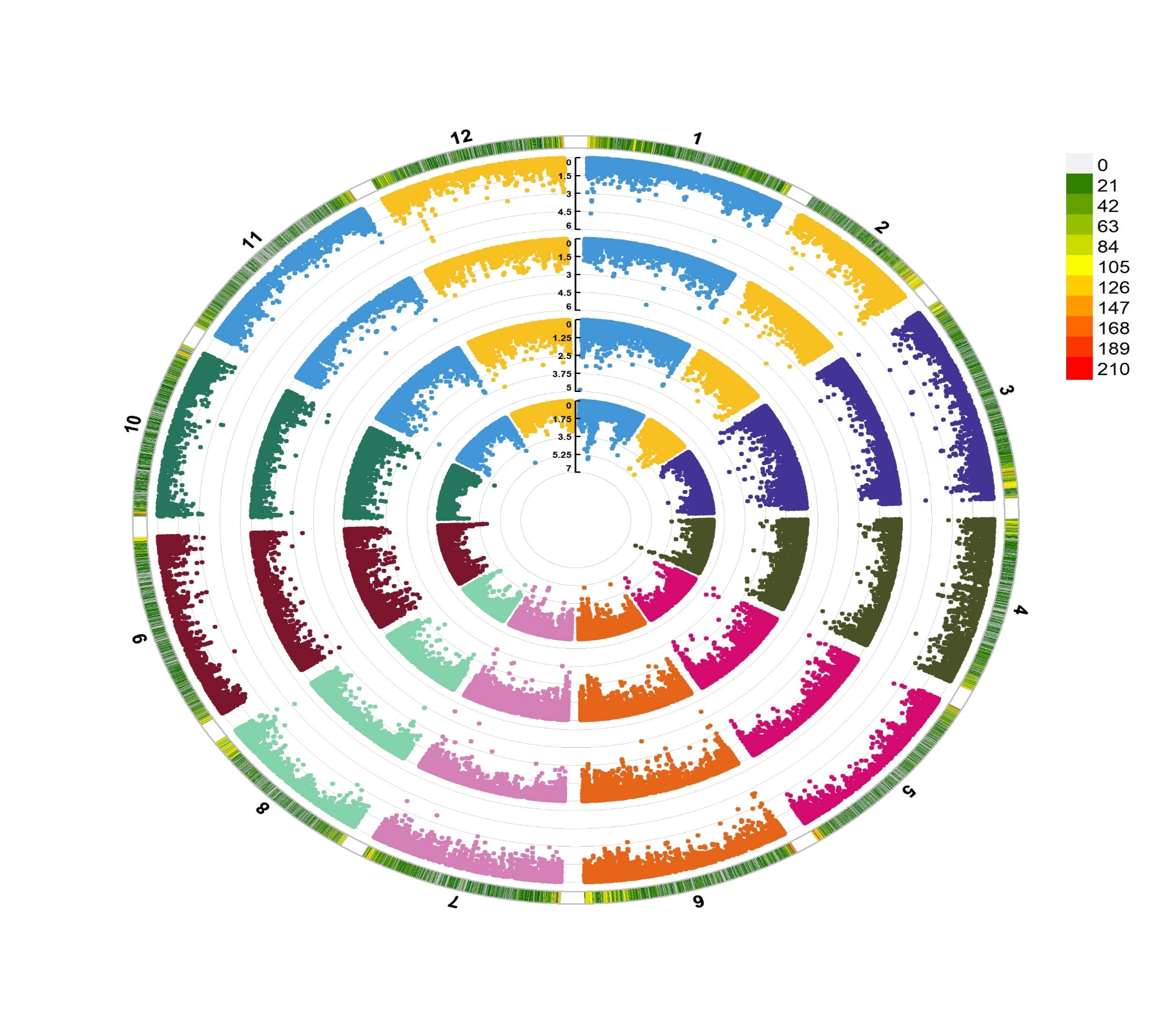


**Figure S5**. Manhattan plot showing SNPs significantly associated with the Carotenoid traits in A) α-carotene, B) β-carotene, C) Capsanthin, and D) Zeaxanthin in *C. chinense*. Different colors on the x-axis indicate 12 chromosomes of the pepper genome. Values on the y-axis are P values of -log 10.

**
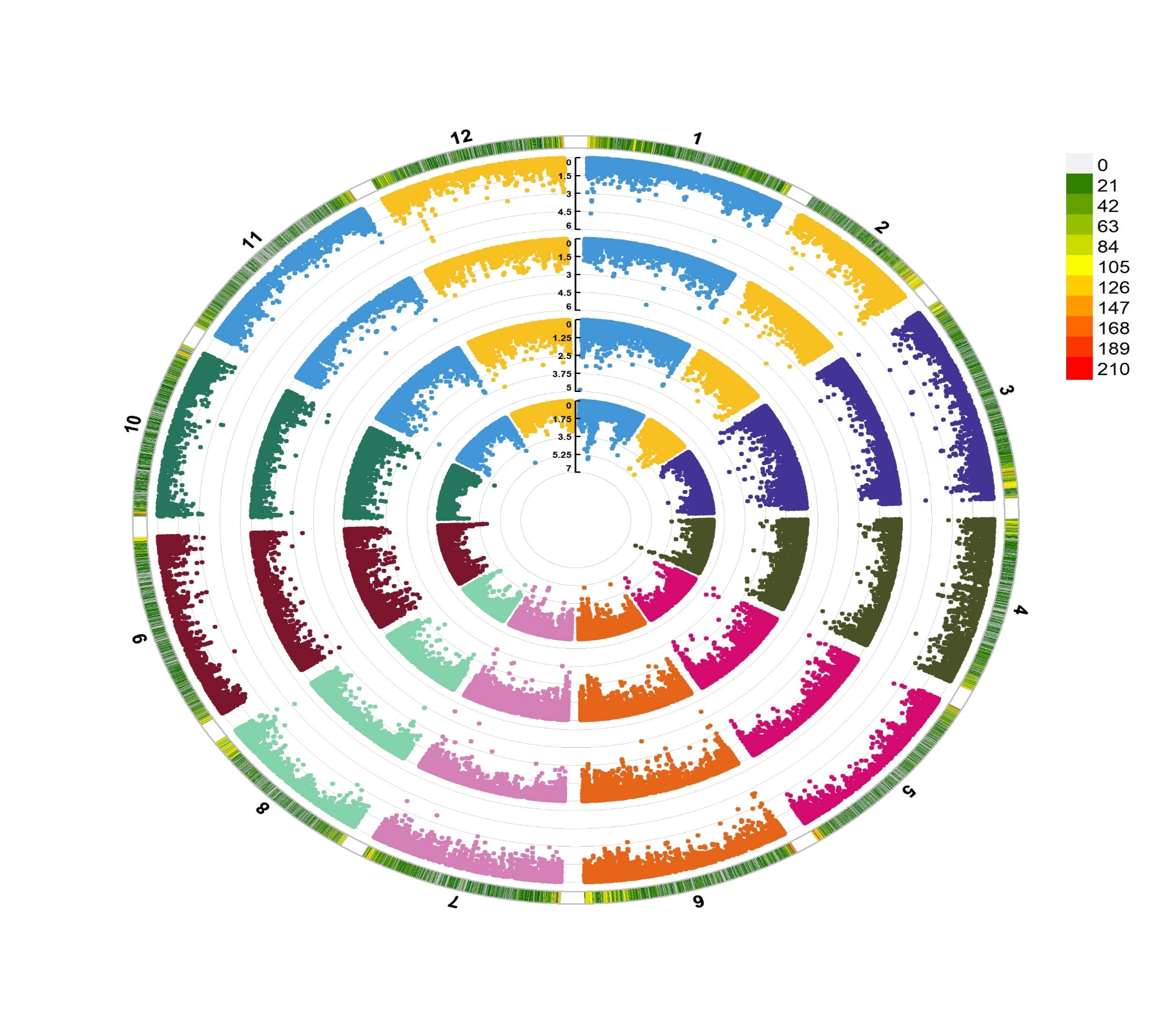
**

**Figure S6**. Manhattan plot showing SNPs significantly associated with the Flavor traits, A) 4-Methylpentyl 4-methylpentanoate, 4-Methylpentyl 3-methylbutanoate, 4-Methylpentyl 2-methylbutanoate, 2-Hexanal in *C. chinense*. Different colors on the x-axis indicate 12 chromosomes of the pepper genome. Values on the y-axis are P values of -log _10_.

**A)**

**
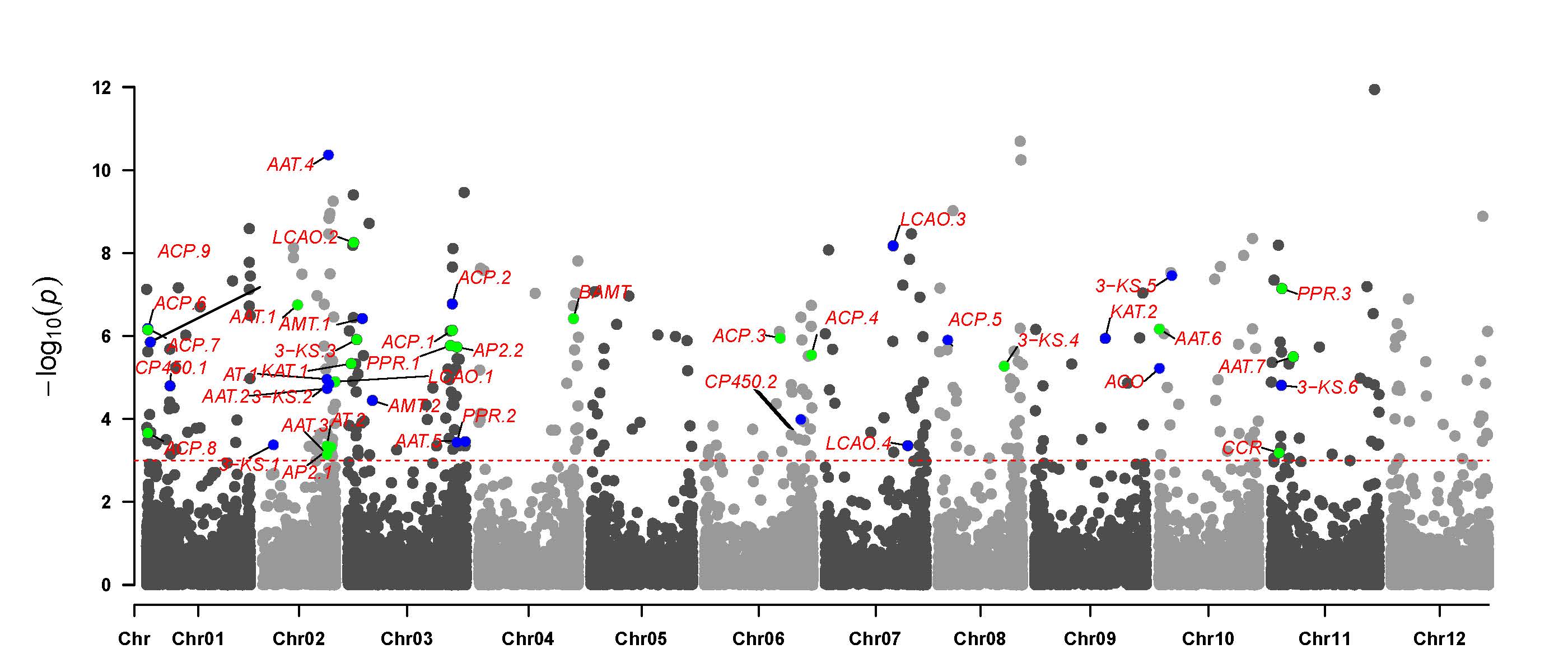
**

**B)
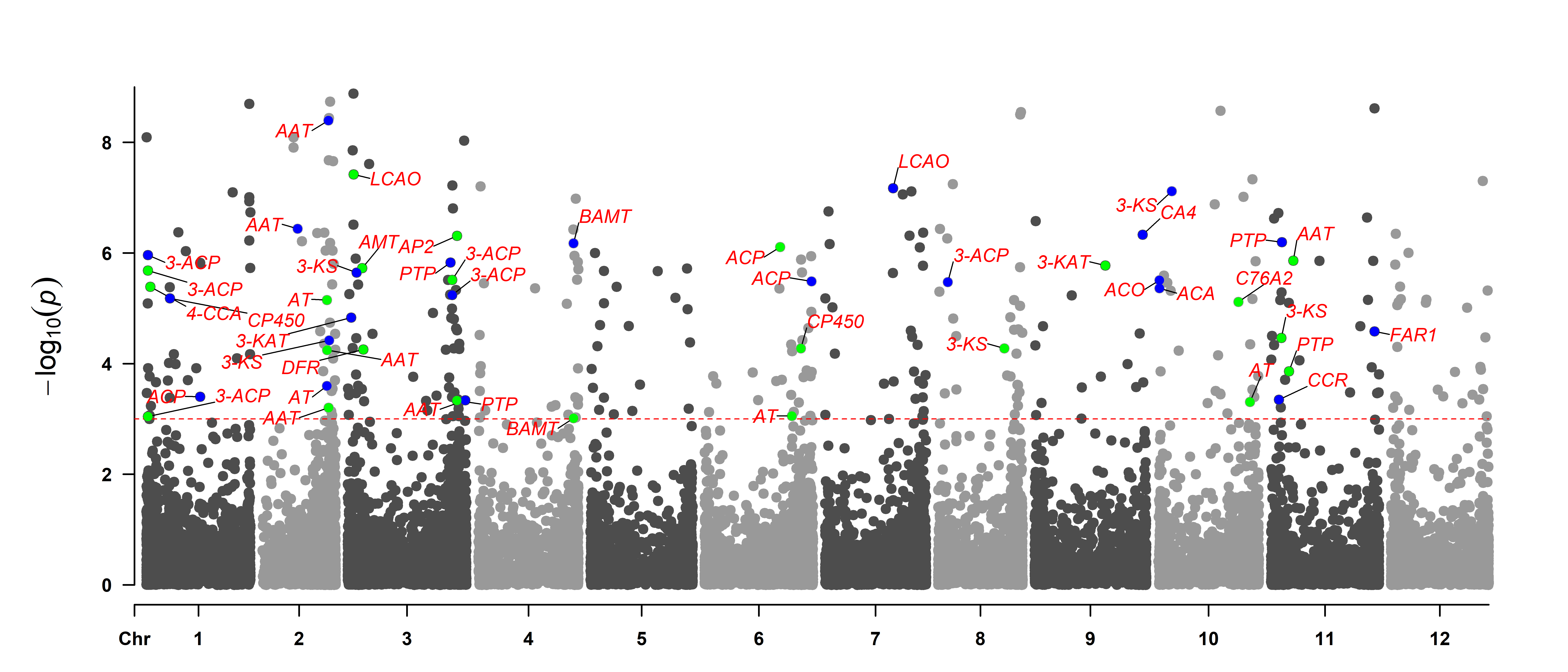
**

**Figure S7**. Manhattan of TWAS for A) Capsaicin and B) DCA. Pathway and regulatory genes were highlighted genes in plots.

**
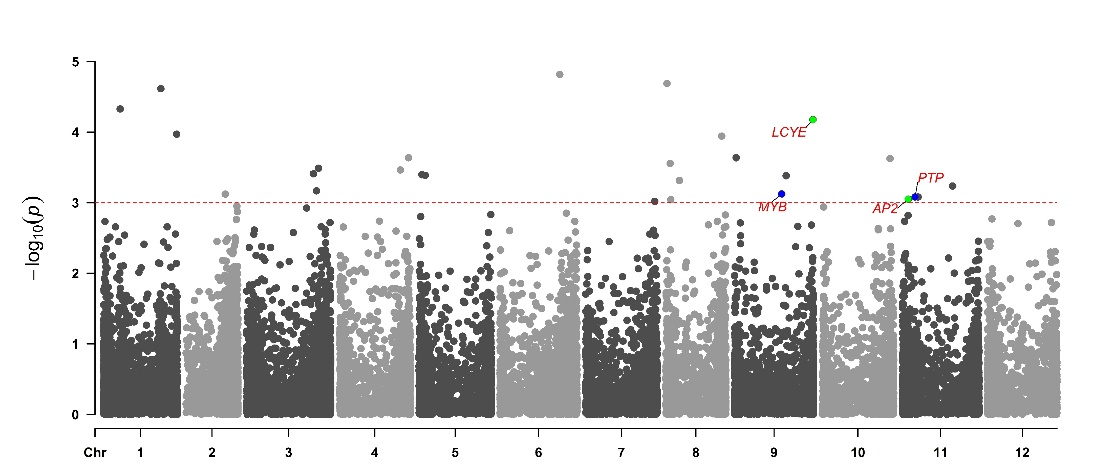
A)**

**
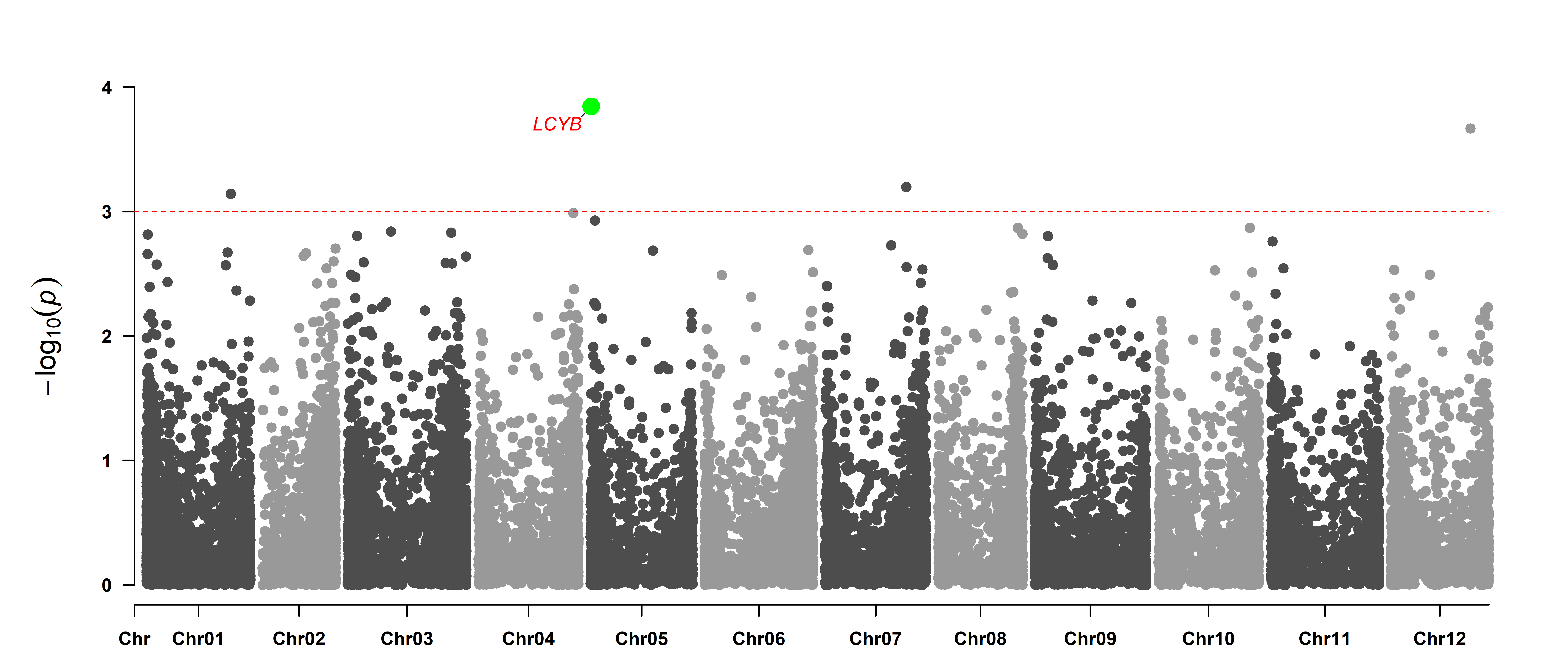
B)**

**
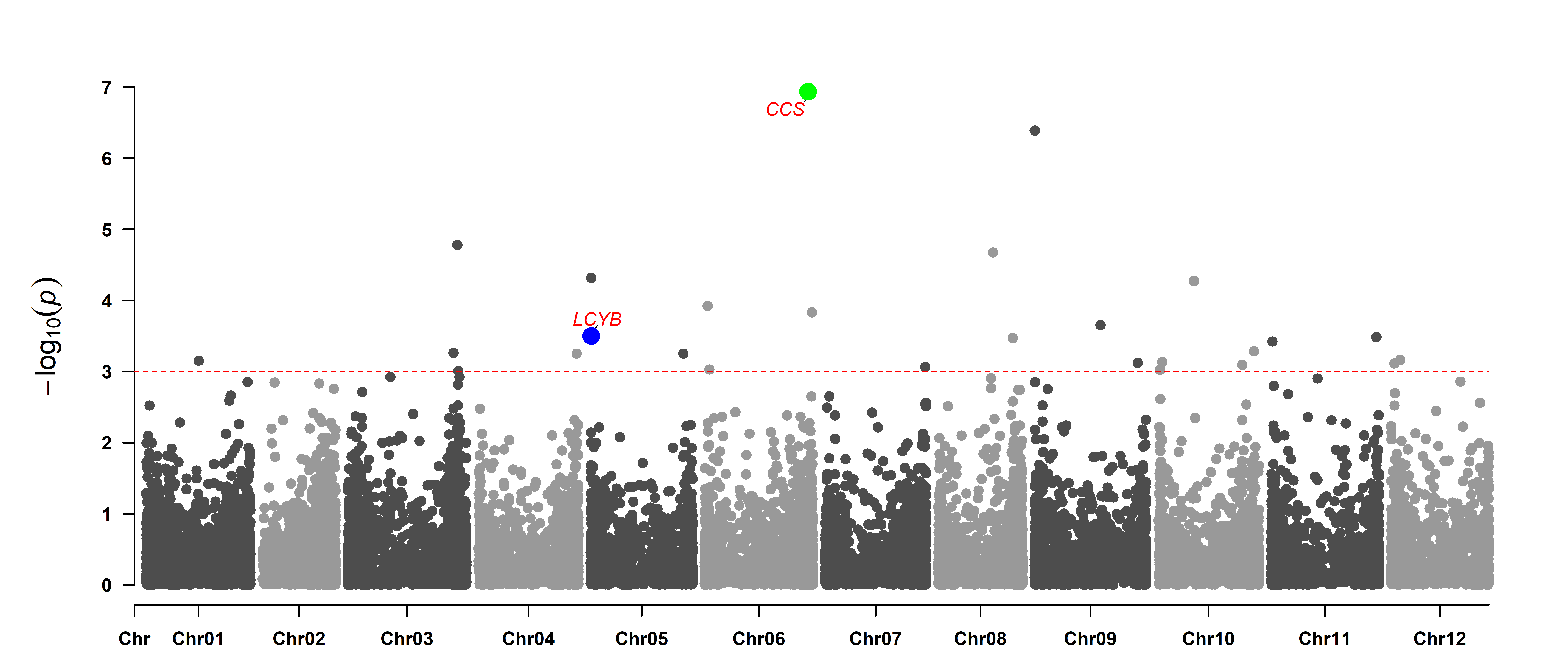
C)**

**
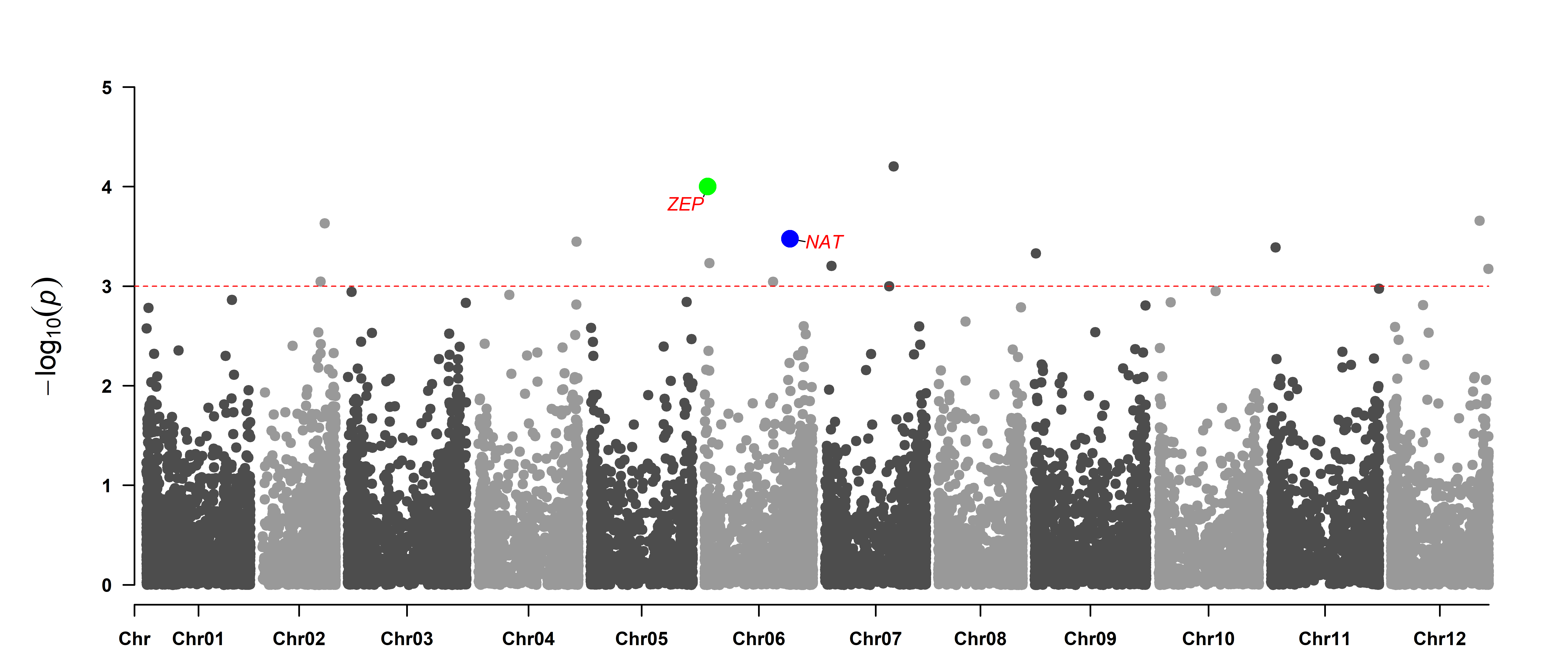
D)**

**Figure S8**. Manhattan of TWAS A) α-Carotene, B) β-Carotene and C) Capsanthin, D) Zeaxanthin. Pathway and regulatory genes were highlighted genes in plots.

**
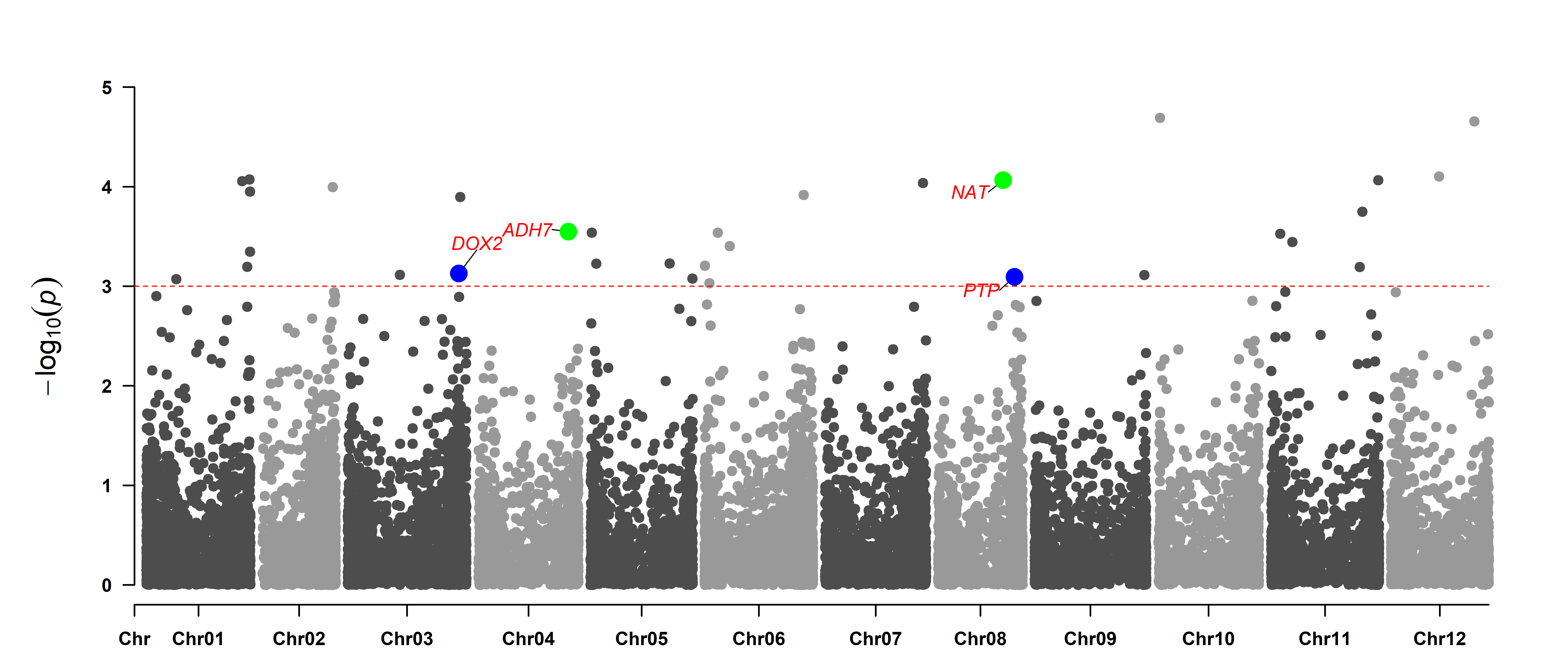
A)**

**
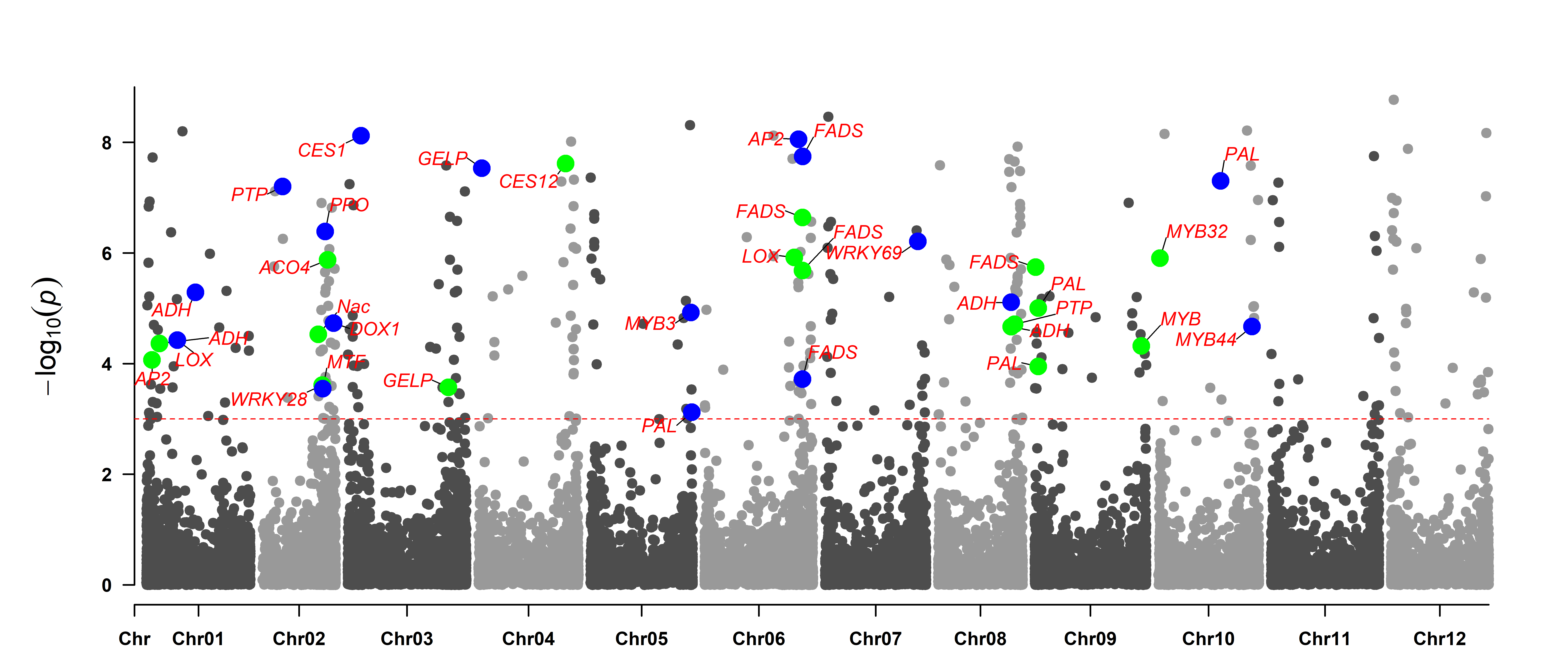
B)**

**
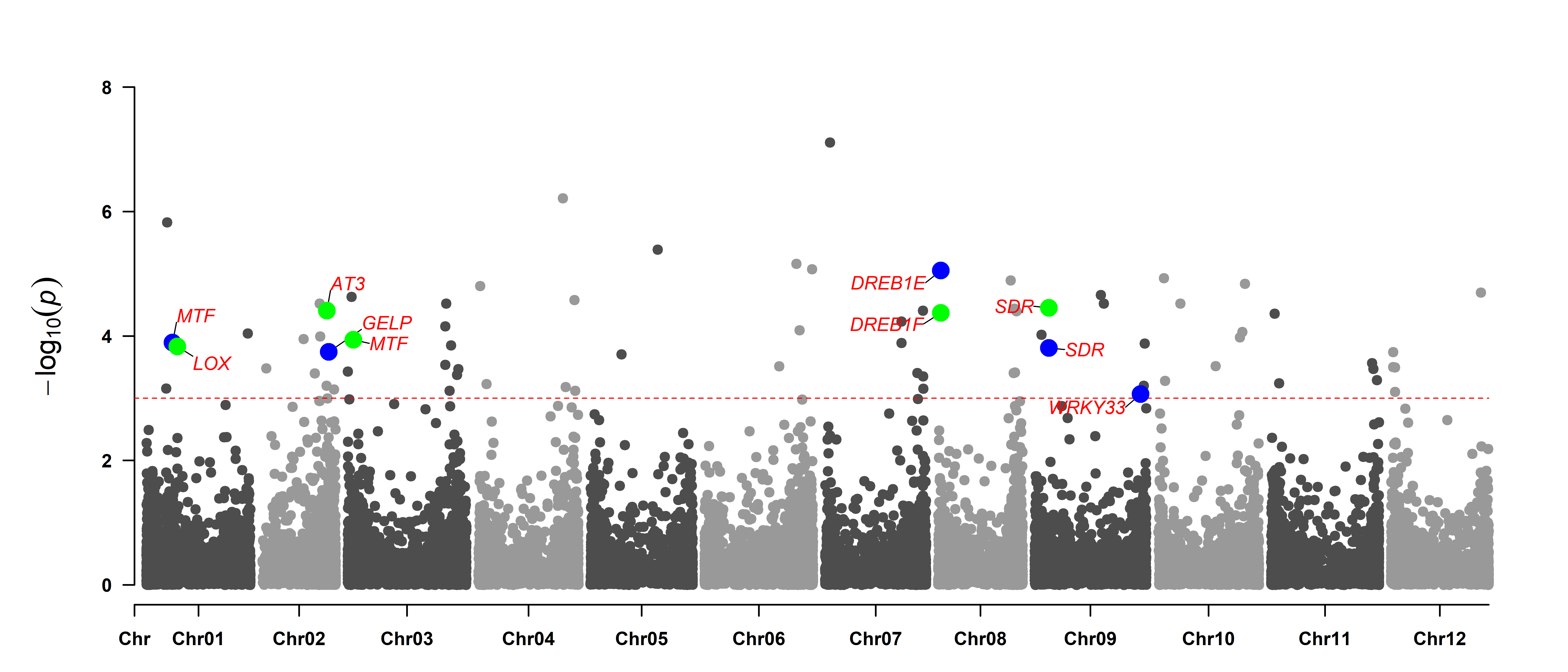
C)**

**
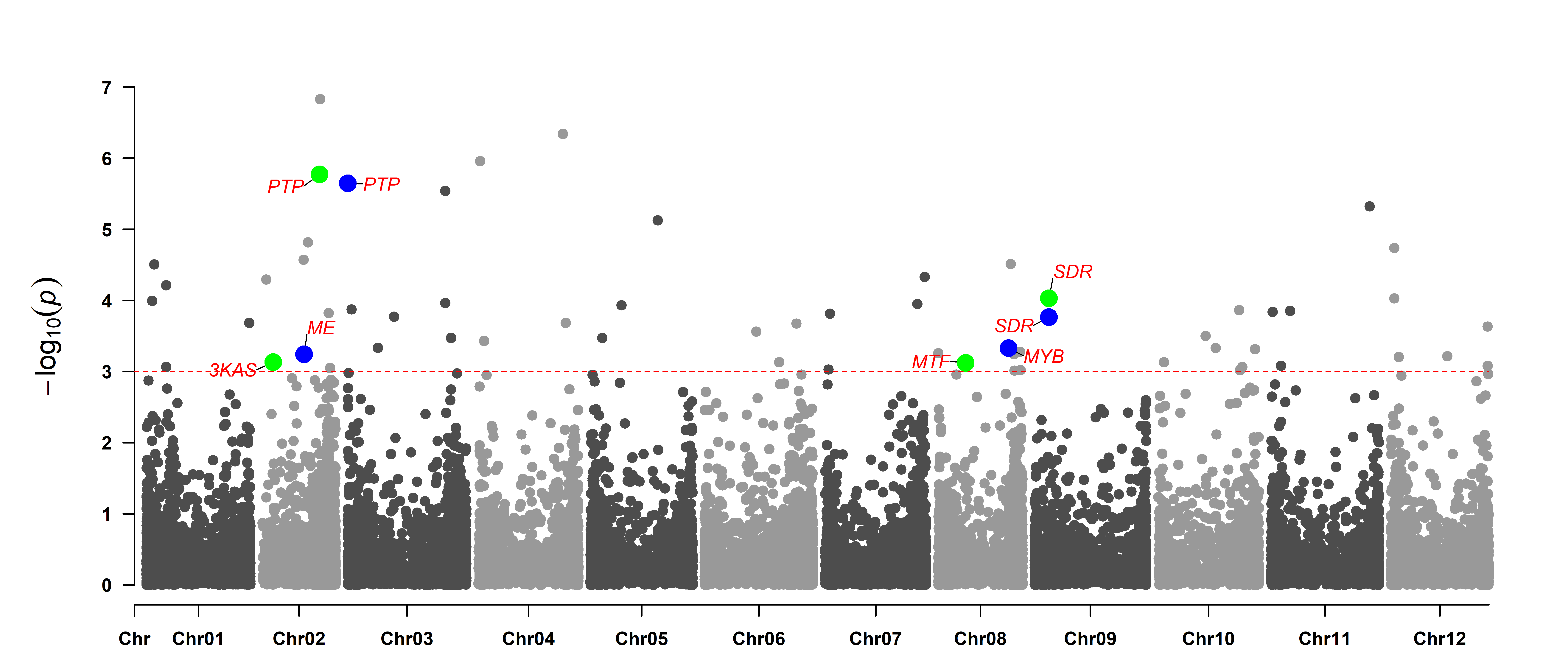
D)**

**Figure S9**. Manhattan of TWAS A) 2-Hexanal, B) 4-Methylpentyl-4-methylpentanoate, C) 4-Methylpentyl-3-methylbutanoate and D) 4-Methylpentyl-2-methylbutanoate. Pathway and regulatory genes were highlighted genes in plots.

**
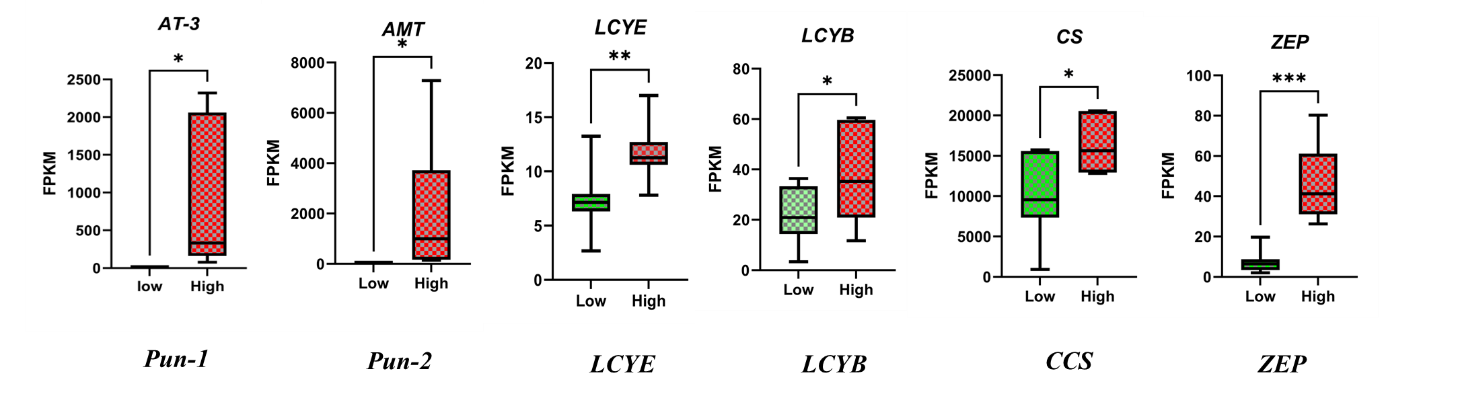
**

**Figure S10**. Expression of *Pun-1, Pun-2, LCYE, LCYB, CCS* and *ZEP* in fruit samples between contrasting phenotypes is shown. ns, not significant; *p % 0.1, **p % 0.01, as determined by Welch’s two-sample single-tailed t-test.


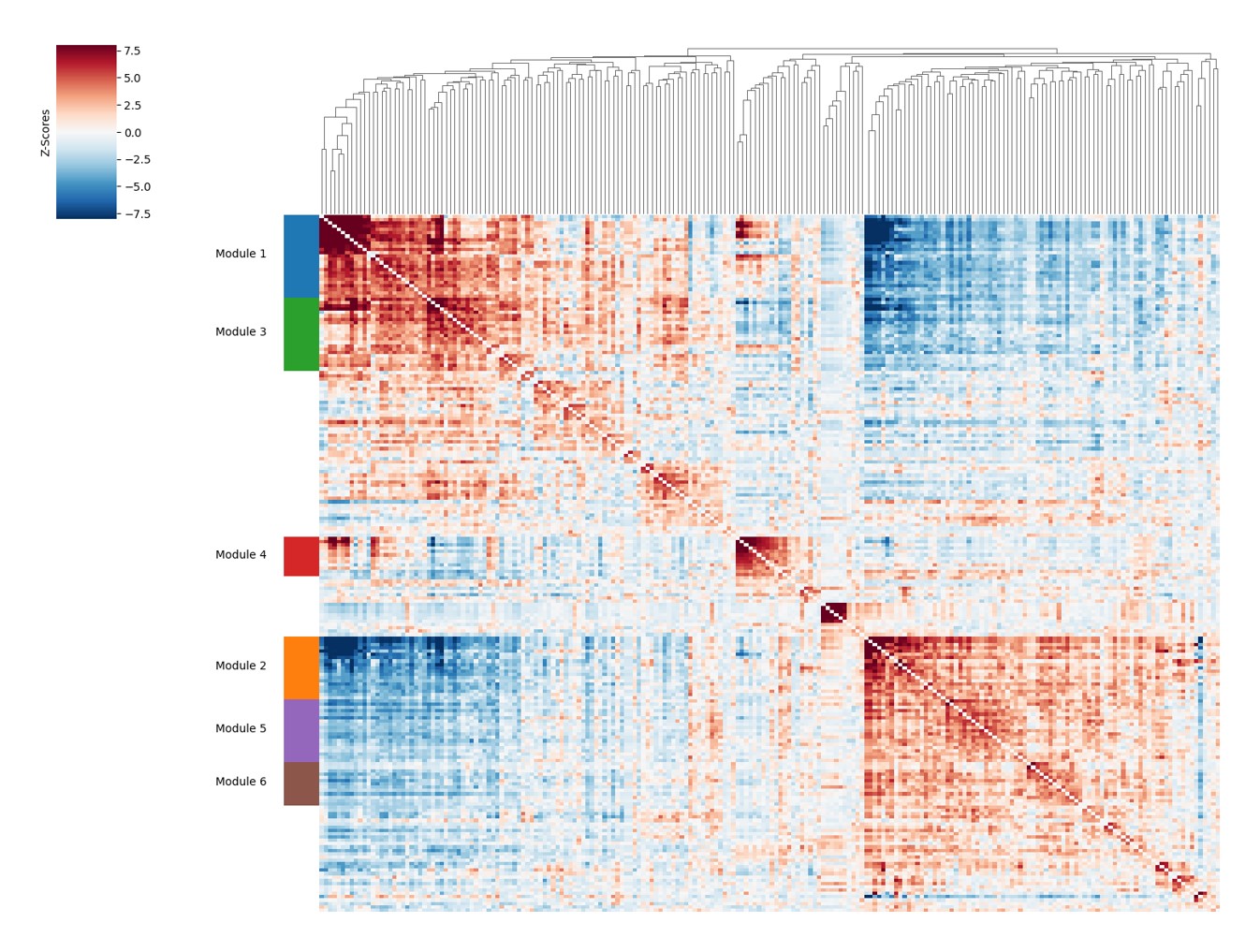


**Figure S11**. The top informative genes selected by Hotspot are grouped into six modules based on pairwise local correlation.


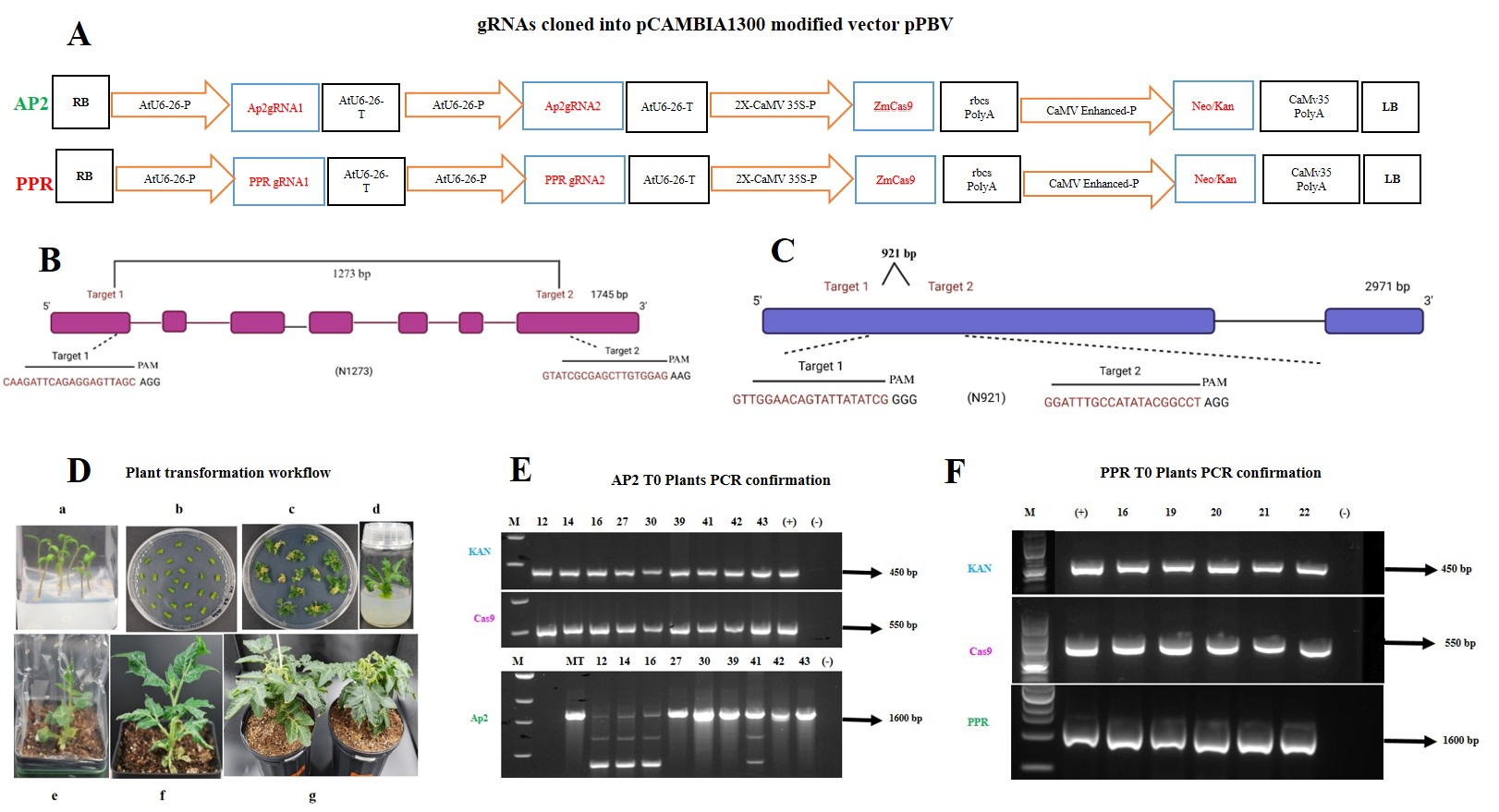


**Figure. S12**: gRNAs cloning into pCAMBIA modified vector pPBV of AP2 and PPR. **B.** AP2 gene structure and gRNA targeted regions. **C.** PPR gene structure (boxes- exons and lines indicate introns; and dot lines are gRNA targeted regions). D. Plant transformation workflow; **a.** eight-day-old cotyledons **b.** Transformed explants in preculture the media. **c.**Regenerated shoots on kanamycin selection **d.** complete plant **e.** Acclimatizated plants **f.g**.. Confirmed transgenics. E. *AP2* transgenic confirmation with kanamycin, cas9, and gene specific primers, respectively. F. PPR transgenic confirmation with kanamycin, cas9, and gene-specific primers, respectively.  **M**: Marker (1 kb ladder); (**+**): positive control; (**-**): Negative control; **MT**: micro tom.
